# The Chromosome-level genome of *Aesculus wilsonii* provides new insights into terpenoid biosynthesis and *Aesculus* evolution

**DOI:** 10.3389/fpls.2022.1022169

**Published:** 2022-10-18

**Authors:** Lichun Ye, Lulu Yang, Bo Wang, Gang Chen, Liping Jiang, Zhigang Hu, Zhaohua Shi, Yifei Liu, Shilin Chen

**Affiliations:** ^1^ College of Pharmacy, Hubei University of Chinese Medicine, Wuhan, China; ^2^ Genomics Project Department, Wuhan Benagen Tech Solutions Company Limited, Wuhan, China; ^3^ Hubei Institute for Drug Control, Wuhan, China; ^4^ Department of Pharmacy, Wuhan Hospital of Traditional and Western Medicine, Wuhan, China; ^5^ Key Laboratory of Chinese Medicine Resource and Compound Prescription, Ministry of Education, Hubei University of Chinese Medicine, Wuhan, China; ^6^ Institute of herbgenomics, Chengdu University of Traditional Chinese Medicine, Chengdu, China; ^7^ Institute of Chinese Materia Medica, China Academy of Chinese Medical Sciences, Beijing, China

**Keywords:** *Aesculus wilsonii*, chromosome-level genome, whole-genome duplication, terpenoid biosynthesis, aescins, phylogenetic relationship

## Abstract

*Aesculus* L. (buckeye and horse chestnut) are woody plant species with important horticultural and medicinal values. *Aesculus* seeds are widely used as biomedicine and cosmetic ingredients due to their saponins. We report a chromosomal-scale genome of *Aesculus wilsonii*. Sequences amounting to a total of 579.01 Mb were assembled into 20 chromosomes. More than half of the genome (54.46%) were annotated as repetitive sequences, and 46,914 protein-coding genes were predicted. In addition to the widespread gamma event with core eudicots, a unique whole-genome duplication (WGD) event (17.69 Mya) occurred in *Aesculus* after buckeye differentiated from longan. Due to WGD events and tandem duplications, the related synthetic genes of triterpene saponins unique to *Aesculus* increased significantly. Combined with transcriptome characterization, the study preliminarily resolved the biosynthetic pathway of triterpenoid saponins like aescin in *A. wilsonii* genome. Analyses of the resequencing of 104 buckeye accessions revealed clear relationship between the geographic distribution and genetic differentiation of buckeye trees in China. We found that the buckeye species found in southern Shaanxi is *A. wilsonii* rather than *A. chinensis*. Population dynamics analysis further suggests that the population size and evolution of existing buckeye species have been influenced by climate fluctuations during the Pleistocene and recent domestication events. The genome of *A. wilsonii* and population genomics of *Aesculus* provide a resource for future research on Hippocastanaceae. These findings will contribute to the utilization and diversity protection of *Aesculus*.

## Introduction

Buckeye trees (*Aesculus*, Hippocastanaceae, Sapindales) are important medicinal and ornamental plants (Barton and Castle, 1877). In the Pharmacopoeia of the People’s Republic of China (2020 ed.), the seeds of *A. wilsonii*, *A. chinensis*, and *A. chekiangensis* are reported to be sources of traditional Chinese medicine (TCM) such as Semen Aesculi (*Suo Luo Zi*), which is used for treating dyspnea, abdominal distention, and epigastralgia (Commission, C.P, 2020). In Europe, the seeds and the bark of young branches of *Aesculus hippocastanum* (horse chestnut) are also widely used as medicines (Braga et al., 2012; Idris et al., 2020). The therapeutic properties of *Aesculus* seeds were first recorded in *Zhou Hou Bei Ji Fang*, which was written by Ge Hong during the Eastern Jin Dynasty in China to treat abdominal pain and for detoxification (Ge, 1963). In the *Compendium of Materia Medica* (*Bencao Gangmu*), *Aesculus* seeds were described as sweet-warm and non-toxic, and recommended for treatment of rheumatic hands and foot spasms (Li, 1982). The chemicals of Semen Aesculi include triterpenoid saponins, flavonoids, and lipids. The aescins with the oleanane type triterpenoid saponins are the main active ingredients in seed (Zhang et al., 2010), with anti-ischemic edema, anti-inflammatory, and anti-tumor pharmacological properties (Cheong et al., 2018; Gallelli, 2019).

Triterpene saponins are widely present in the normal growth and development of various plants. Previous studies have paid wide attention on the biosynthesis of triterpenoid saponins in plants (Thimmappa et al., 2014). Triterpenoid saponins are typically synthesized through the isoprenoid pathway, and cyclized by of 2,3-oxidosqualene to give oleanane (β-amyrin) or dammarane triterpenoid skeletons (Haralampidis et al., 2002). A variety of oxidosqualene cyclases (OSCs) are charge for the cyclization step, which marks the branch point in the metabolism of primary and secondary triterpenes. The triterpenoid backbones are then modified by various cytochrome P450-dependent monooxygenases (CYPs), UDP-glycosyltransferases (UGTs), acyltransferases, methyltransferases, and others to increase the complexity, expand the structure and functional diversity (Thimmappa et al., 2014). However, knowledge of the relationships of enzymes and biochemical pathways involved in saponin biosynthesis remains limited. Novel functionalization of a cellulose synthase-like (Csl) enzyme with a form of triterpenoid glycosyltransferase activity enables glucuronic acid attach to the C-3 position of saponins like aescins (Jozwiak et al., 2020). The major aescins (aescin1a, aescin1b, isoescin 1a, isoescin 1b) in the seeds are the parent nucleus of oleanolic acid with three glucose-forming glycoside substitutions at the C3 position. The subtle different between aescins were substituted by cis-trans ethylenic configuration at C21 position, and whether the C22 and C28 positions were substituted by acetyl (Colson et al., 2019). Little has been reported about the biosynthetic pathway of aescins due to the lack of reliable genomic information.

Approximately 12–19 species of *Aesculus* have been identified in eastern Asia, eastern and western North America, and southeastern Europe (Hardin, 1957a; Hardin, 1957b; Hardin, 1957c). Based on morphological and DNA sequence data from ITS, *rbcL*, and *matK*, all *Aesculus* species represent a monophyletic group that can be further divided into five sections. The *Calothyrsus* section includes species that mainly occur in eastern Asia (Xiang et al., 1998; Harris and Thomas, 2009; Du et al., 2020). Three TCM species (*A. wilsonii*, *A. chinensis*, and *A. chekiangensis*) and *Aesculus wangii* from Yunnan are relatively widespread taxa in this section. They are closely related and have very similar morphologies (FANG, 1981). For example, cultivated buckeye plants from southern Jiangsu and northern Zhejiang are described as *A. chekiangensis*, which has been considered to be a variety of *A. chinensis* due to its very similar morphology (Xia and Turland, 2005). Introgression or hybridization is also possible given that numerous intermediates between *A. wilsonii* and *A. chinensis* occur within the natural range of *A. wilsonii* (Hardin, 1957c). Clarifying the genetic relationship of buckeye species in China is therefore important for the optimization of their medicinal applications.

**Figure 1:**
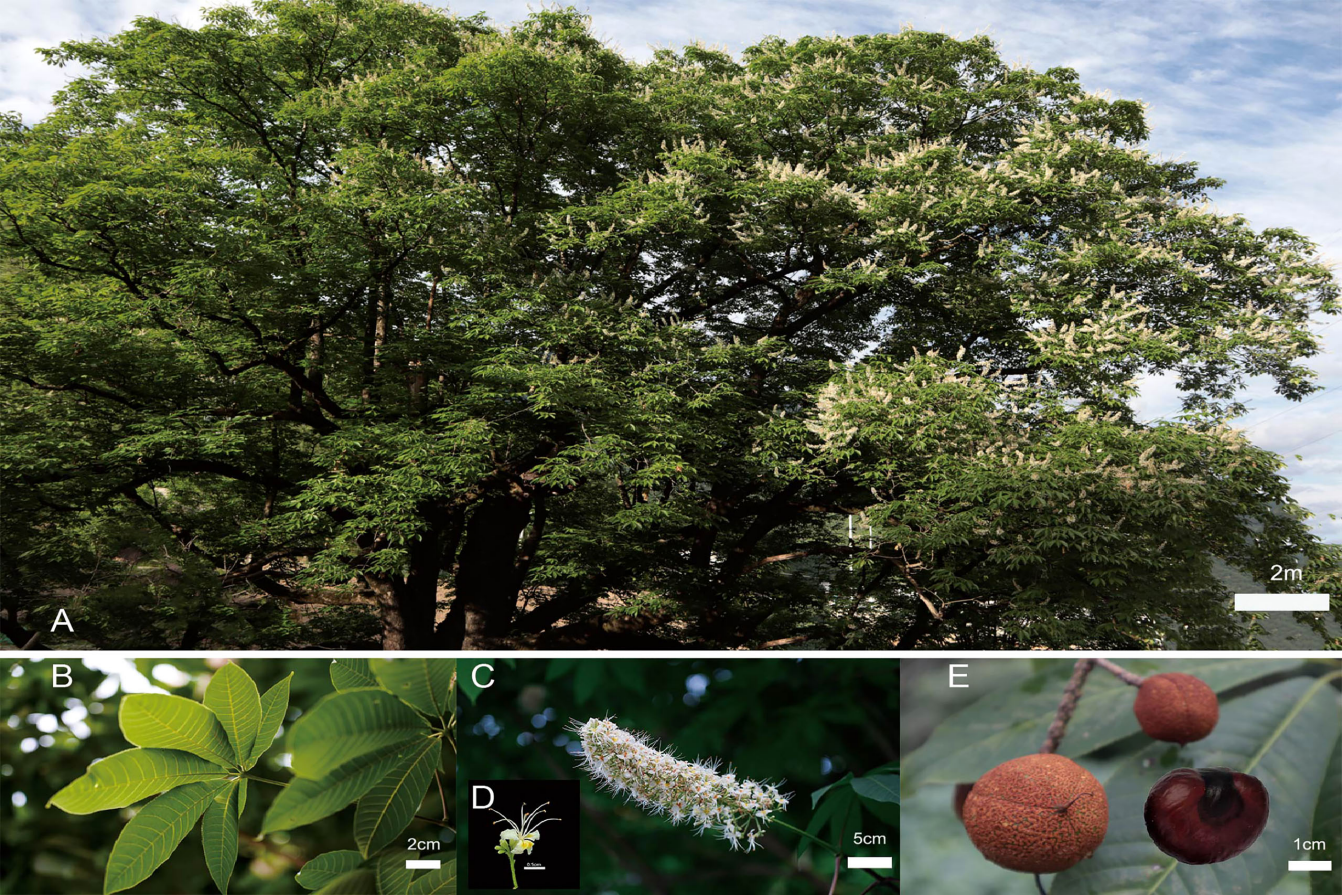
Morphological characteristics of *Aesculus wilsonii.* **(A)** A mature *A*. *wilsonii* tree growing in Shennongjia National Nature Reserve. **(B)** Mature leaves of *A*. *wilsonii*. **(C, D)** Flowers. **(E)** Fruits and seed, Bar=1cm.

In this study, we sequenced and assembled a chromosome-scale genome of *A. wilsonii* (**Figures 1A–E**). We performed a phylogenetic and comparative genome analysis to investigate the phylogenetic position of *A. wilsonii* within representative flowering plants and also the ancient whole-genome duplication (WGD) events occurring during *A. wilsonii* evolution. With available genome data, the main gene families and genes related in the biosynthesis of triterpenoid saponin were also examined, and their expression in different tissues were documented. Resequencing of population samples of *A. wilsonii* and *A. chinensis*, *A. chekiangensis*, and *A. wangii* in China were also conducted to clarify their genetic relationships and evolutionary demography. The results provide a reference for the resource conservation and further utilization of *Aesculus* plants.

## Materials and methods

### Plant materials

The *A. wilsonii* tree used for genome sequencing was from Shennongjia National Nature Reserve, Hubei, China. Fresh young leaves were collected and immediately frozen for next DNA extraction. A total of 104 resequencing *Aesculus* samples were collected from 17 populations from six provinces, representing four different species including *A. wilsonii*, *A. chinensis*, *A. chekiangensis*, and *A. wangii* (**Supplementary Table 1**).

### Genome library construction and sequencing

By the modified CTAB method (Porebski et al., 1997), we extracted high-quality genomic DNA from young leaves, and checked the quality and quantity of the isolated DNA by A260/A280 nm and 0.5% agarose gel electrophoresis. A paired-end library with short-insert (300–350 base pairs) was constructed following manufacturer protocol (Illumina). To ensure the library quality, Agilent 2100 and q-PCR were used to detect the inserted fragments, and accurately quantify the effective concentration of the library. Quality-compliant libraries were sequenced using the Illumina NovaSeq platform. The genomic DNA was purified and fractionated by the BluePippin system (Sage Science) and then used to construct the sequencing libraries by the protocol provided with the SQK-LSK109 genomic sequencing kit (Oxford Nanopore Technologies) for the Oxford Nanopore MinION platform (ONT).

### Hi-C sequencing

The DNA of young leaves was extracted after fixing chromatin with formaldehyde for Hi-C sequencing. Qualified samples were digested by restriction endonuclease. The fragments were purified after labeling the biotin nucleotides and ligating the blunt ends to each other. The Hi-C fragment was processed by removal of biotin, ultrasound interruption, terminal repair, addition of base A and PCR amplification to construct the library. A 150-bp paired-end mode was used to sequence the library on an Illumina NovaSeq System.

### RNA sequencing

The total RNA of the flowers, leaves, seeds of *A. wilsonii* were extracted using the R6827 Plant RNA Kit following manufacturer instructions. After the RNA samples were qualified, the entire library was prepared by random interruption, end repair, A-tail addition, sequencing adapter addition, purification, and PCR amplification. After the libraries were qualified, according to the requirements of effective concentration and target data volume, the different libraries were pooled to the Flow cell and sequenced by the Illumina NovaSeq platform. The raw data was filtered by fastp (ver.: 0.21.0; parameter: default) to obtain clean data, and we aligned the filtered transcriptome sequence with the reference genome according to Star (version: 2.7.9a; parameter: default). We aligned the number of reads of each transcript for each sample by RSEM, and performed fragments per kilobase per million bases (FPKM) calculation to obtain the expression levels of genes and transcripts.

### Genome survey and assembly

The genome size and heterozygosity of *A. wilsonii* were estimated with short reads by *k-mer* analysis. With GCE and Genome Scope tool (Liu et al., 2013), the genome size was estimated by dividing the total number of *k-mers* counted by the *k-mer* coverage based on the 19-mer frequency of Illumina short reads, and the first estimate of the GC content, heterozygosity, and repeat content. The clean long reads were filtered by MinKNOW from the raw fastq data of ONT sequencing were corrected and assembled by SMART *de novo* (Schmidt et al., 2017) with default parameters. Racon (Vaser et al., 2017) (Version:1.4.11) software was used to conducted two rounds of polishing based on third-generation sequencing data, and two rounds were polished with Pilon (Walker et al., 2014)(version:1.23) by Illumina short reads. The map_rate with Illumina reads, coverage were used to estimate the quality of the genome assembly by the BUSCO (Simão et al., 2015) (v.3.0.1). The Hi-C clean data were aligned to the preceding scaffold assembly by using BWA software with default parameters (Li and Durbin, 2009). SAMTOOLS (Li et al., 2009) was used to remove duplicates (parameters: rmdup) and nonaligned data. Statistical analysis was performed on unique mapped reads, and unique read pairs with interaction effectively around the restriction site were identified to be used to build the pseudo-chromosome sequences. ALLHiC (Zhang et al., 2019) (v0.9.12) was used to construct the scaffold graph by optimizing the ordering and orientation of each clustered group within simple diploid mode. Finally, the scaffolds were anchored to 20 chromosomes, and an interaction heat map of each chromosome heatmap was plotted.

### Genome annotation

We combined the *de novo* and homology-based methods to identify the repeat sequences. The repetitive sequences in the genome of *A. wilsonii* were filtered by RepeatModeler (Flynn et al., 2020) (version:1.0.4) to constructed the repeat library. Then we combed the repeat library and the repbase-derived RepeatMasker library. Based on homology in RepeatMasker (Chen, 2004), the repetitive sequences were identified by a conserved BLASTN search. Non-coding RNAs can also have important biological functions. Based on their structural characteristics, tRNAsan-SE (Lowe and Eddy, 1997)(version1.23) was used to search the tRNAs in the *A. wilsonii* genome, and RNAmmer (version: 1.2) was used to predict the rRNA. According INFERNAL (Nawrocki and Eddy, 2013)(Version: 1.1.2), the ncRNA and snRNAs sequences in the genome were identified based on the Rfam (Ioanna et al., 2018) database. We used MAKER (Kondrashov et al., 2011)(version:2.31.8) combined *de novo* and homology and transcriptome-based predictions, to predict protein-coding genes. The transcriptome results were *de novo* spliced by Trinity (Grabherr et al., 2013) (version: v2.6.6) and annotated as EST data. We used the model of Augustus (Hoff et al., 2016) (tomato) combined with other species including *Solanum lycopersicum*, *Arabidopsis thaliana*, *Vitis vinifera*, *Oryza sativa*, *Dimocarpus longan*, *Citrus sinensis*, *Atalantia buxifolia*, *Citrus reticulata*, *Citrus grandis* and *Helianthus annuus* for genetic structure prediction. The functional annotation of all *A. wilsonii* genes was performed by homologous alignments with BLASTP (version:0.7.9,version,e-value ≤1e−5) in public protein databases such as Uniprot (https://www.uniprot.org/), NR, KEGG (Minoru et al., 2014), InterProScan (Zdobnov and Rolf, 2001) (version: 5.33–72.0), and Pfam (Finn et al., 2008).

### Genome evolution

Ten other species—*A. buxifolia, D. longan, C. reticulata, C. grandis, C. sinensis, C. nankingense, H. annuus, A. thaliana, V. vinifera*, and *O. sativa*—were selected for identifying the gene family of *A. wilsonii* by OrthoMCL (Li et al., 2003). We aligned 1521 single-copy genes that were common to the 11 selected genomes using MUSCLE (Edgar, 2004) (version: V3.8.31). alignment results were filtered (-GT 0.2) by trimal (version: V1.4.rev 22). Then the phylogenetic tree with PROTGAMMAWAG model was constructed by RAxML (Kevin et al., 2011) (Version: 8.2.10). CAFÉ (Bie et al., 2006) (version: 2.1; Parameter: – filter) was used for gene family contraction and expansion analysis. Using referenced the fossil nodes were selected from TimeTree (Sudhir et al., 2017) (http://www.timetree.org/), MCMCtree was applied to estimate the divergence time of 11 species.in PAML (Yang, 2007) (version 4.9). The Ka/Ks ratios of genes calculated by the PAML package were used to detect the positive selection genes with a threshold value of Ka/Ks ≥ 1 and a P value ≤.05.

### Synteny and genomic duplication analysis

Whole-genome duplication (WGD) events are important for the adaptive genome evolution of species. The protein sequences of different species were matched using BLAST (version: 2.6.0+, parameters: e-value 1–5 - e outfmt 6), then MCScanX (Tang et al., 2008) (https://github.com/wyp1125/MCScanx; Parameters: -a - E 1E-5-S 5) were used to analyze collinear blocks of the intra- and interspecies comparisons of the *A. wilsonii* genome. PAML (version: 4.9, yn00) was used to calculate the synonymous mutation frequency (Ks), non-synonymous mutation frequency (Ka), and the ratio of non-synonymous mutation rate to synonymous mutation rate (Ka/Ks) of collinear gene pairs, and they were plotted using GGploT2 (version: 2.2.1). According to T=Ks/2r, the divergence time was converted by calculated Ks value with a substitution rate in eudicots was 6.5×10-9 mutations per site per year.

### Genes related to pentacyclic triterpenoid saponins biosynthesis

Pentacyclic triterpenoid saponins are the main medicinal component of *A. wilsonii*. We retrieved protein sequences of the terpenoid backbone, sesquiterpenoid and triterpenoid biosynthesis pathway (00900,00909) from *Glycine max* and *Arabidopsis thaliana*, including ACAT, HMGS, HMGR, MVK, PMK, MVD, IDI, DXS, DXR, MCT, CMK, MDS, HDS, HDR, FPPS, GPPS, GPS, SQS, and SQE from the NCBI database. Based on these homologs as queries, we identified the candidate protein sequences for homologs to these proteins in the *A. wilsonii* genome. The OSC, CYP450, BAHD, SCPL, Csl, and UGT genes were predicted using hmmsearch according the OSC hmm model (PF13249 and PF13243), the CYP450 hmm model (PF00067), BAHD hmm model (PF02458), SCPL hmm model (PF00450), Csl hmm model (PF03552), and UGT hmm model (PF00201) from Pfam and homolog-based BLAST with an e-value cutoff of 1e−5. Using Mega-X (Kumar et al., 2018) (V10.0.5), phylogenetic trees of each gene families related biosynthesis of triterpenoid saponins were constructed from *A. thaliana* and *A. wilsonii*. Then according to the reported reference, the predicted OSC, CYP450, BAHD, SCPL, Csl, and UGT candidates were further divided into clans or families with similar functions.

### Resequencing and quality control

DNAs from leaf tissue of 104 buckeye accessions were isolated by the modified CTAB method. After control of the quality and quantity by 0.5% agarose gel electrophoresis and A260/A280 nm, 104 DNA samples were used for the construction of the libraries on the Illumina NovaSeq 6000 with an expected target coverage of 5×. In total, 369.33 Gb of clean bases were obtained. The clean reads were aligned to the reference genome by BWA (Li and Durbin, 2009)(version:0.7.17, Parameters: mem To obtain high quality SNP, GATK (version: 4.2.0.0, parameter: VariantFiltration) was used to filter the SNP following the officially recommended standard (QD < 2.0 || QUAL < 30.0 || SOR > 3.0 || FS > 60.0 || MQ < 40.0 || MQRankSum < −12.5 || ReadPosRankSum < −8.0).

### Analysis of population structure

Based on the results following GATK hard-filtering, vcftool (Danecek et al., 2011) (version: 0.1.16, parameters: – maf, – max-missing) was used to eliminate minor allele frequencies less than 0.05. The final SNPs of 104 samples were performed to construct a neighbor-joining phylogenetic tree by PHYLIP (Purcell et al., 2007) (version 3.696), and implemented visualization within ggtree (Yu, 2020) based on newick tree file. To illustrate the population genetic structure of buckeye, ADMIXTURE (version: 1.3.0; -cv input File K) was used to estimate the optimal ancestral population from a multi-locus SNP genotype dataset. When the number of subgroups contained in the population was not known, the range of K can be preset from 2 to N. Through a simulation of calculation, the classification of the population and the proportion of each sample’s pedigree can be calculated based on the Bayesian algorithm. We selected the best K value according to error value and maximum likelihood value (Alexander et al., 2009). Based on the degree of variation difference among population samples, GCTA (Yang et al., 2011)(version: 1.93.2, parameters: -GRM, -PCA) was used to cluster individuals into different subgroups according to the PCA method. Based on the probability of simultaneous occurrence of two or more alleles at loci, PopLDdecay (version: 3.26) was used to calculate the R^2^ of the maximum distances of 300 Kb by genome-wide linkage disequilibrium analysis (Vos et al., 2017). The genetic differentiation (*F_ST_*), nucleotide diversity (π), and relatedness PHI were calculated by Vcftools (version: 0.1.16) to analyze the relationships between different subgroups.

### Gene flow and demographic history analysis

To infer the patterns of differentiation and mixing in multiple populations, Treemix (Pickrell et al., 2012) was used to evaluate the gene flow between populations. Based on the genome-wide alleles set, the actual covariance (Real value) between each pair of populations was calculated, and the maximum likelihood trees were constructed to calculate the estimated value of covariance. The difference between the actual and estimated value was used to judge whether gene flow occurs between the two populations. The demographic history of each *Aesculus* population were investigated by the PSMC (Schiffels and Durbin, 2014) with the mutation rate 1.722×10^−9^ per base per generation (Xiang et al., 1998) and the generation time g = 8 years over the last 20 million years.

## Results

### Genome sequencing and assembly

*A. wilsonii* is a diploid (2*n* = 2*×* = 40), with the genome size was estimated to be 552.87 Mb according to *k*-mer (*k* = 19) analysis and a relatively high genome heterozygosity (1.22%) (**Supplementary Table 2**; **Supplementary Figure 1**). The estimated genome size of *A. wilsonii* is very close to those previously reported for other *Aesculus* taxa ranging from 467 to 623.48 Mb (Krahulcová et al., 2017). According Nanopore genome sequencing, a total of 52.11 Gb of raw long reads (**Supplementary Table 3**) were produced. After being filtered, the clean long reads were further self-corrected and polished with 63.76 Gb short reads, and finally assembled to produce a primary genome of 611.22 Mb, including 376 sequence contigs (N50 = 3.75 Mb) (**Supplementary Table 4**).

**Figure 2:**
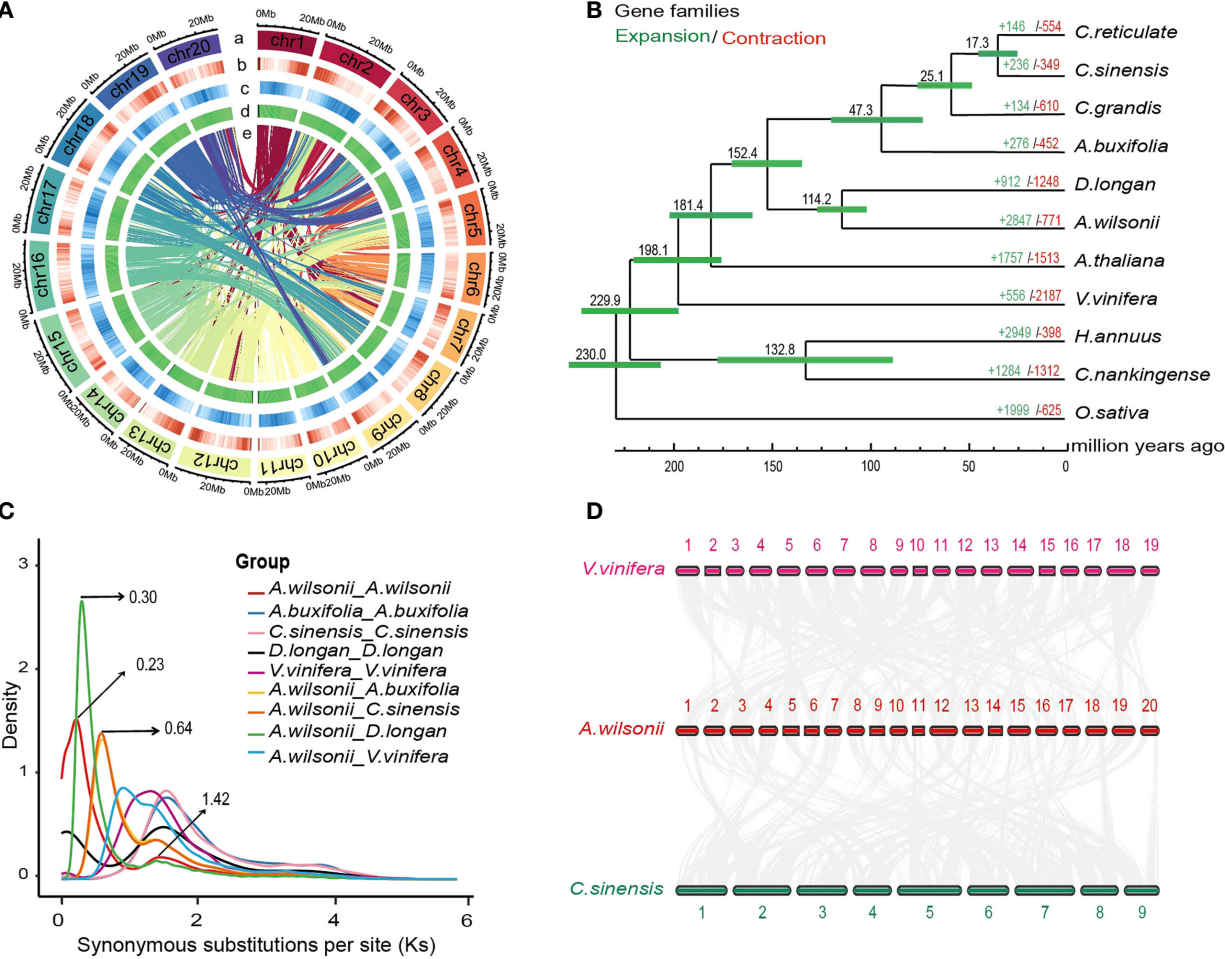
Genome evolution of *Aesculus wilsonii.* **(A)** *Aesculus wilsonii* genomic landscape. a Assembled pseudochromosome, b Gene density, c Repeat density, d GC content, e Syntenic blocks colored lines (from outside to inside). **(B)** The phylogenetic relationships and evolution analysis of *A*. *wilsonii* with other species. Green and red fonts indicate the numbers of expanded and contracted gene families that in 11 species, respectively. The green bars represent the 95% confidence interval of the estimated divergence time. **(C)** Ks distribution of syntenic blocks between *A*. *wilsonii*, *A*. *buxifolia*, *C*. *sinensis, D. longan* and *V. vinifera*. **(D)** Identified synteny blocks between *A*. *wilsonii*, *V. vinifera* and *C*. *sinensis*.

In order to assemble into the chromosome-scale genome of *A. wilsonii*, 221,213,230 read pairs were obtained by Hi-C sequencing, of which 94.72% of reads were mapped to the assembled contigs, including 75,353,070 uniquely mapped read pairs and 29,283,408 read pairs that represented valid interactions (**Supplementary Table 5**). All the mapped contigs were categorized and ordered to construct chromosome-scale scaffolds. Finally, 94.72% scaffolds were anchored to 20 pseudochromosomes (**Figure 2A**; **Supplementary Figure 2**), resulting in a chromosome-level *A. wilsonii* genome assembly of 579.01 Mb (scaffold N50 = 28.02 Mb) (**Supplementary Tables 4, 6**). Further analysis by mapping Illumina short reads showed that about 98.79% reads could be mapped back to the genome assembly (**Supplementary Table 7**). All these collectively suggested that the *A. wilsonii* genome assembly with high quality.

### Genome annotation and repetitive elements

An integrated strategy including the ab initio gene prediction and evidence-based methods were used to annotate the protein-coding of the *A. wilsonii* genome (**Supplementary Figure 3**). After removing nonfunctional annotations, 46,914 protein-coding genes was retained (**Supplementary Table 8**). It was a near completeness of gene prediction with 91% annotated BUSCO gene models were identified by BUSCO software (**Supplementary Table 9**). The 46,914 genes were identified, and 44210 proteins (94.24%) were annotated using four databases of known proteins (**Supplementary Table 10**). In addition, 4282 noncoding RNAs were identified in the *A. wilsonii* genome, including 302 microRNAs, 801 transfer RNAs, 1866 ribosomal RNAs, and 1225 small nuclear RNAs (**Supplementary Table 11**). Approximately 54.46% (332.85 Mb) of the repetitive elements were present in the genome of *A. wilsonii* (**Figure 2A** and **Supplementary Table 12**). Among these, long terminal repeat (LTR) retrotransposons, DNA transposable elements, long interspersed nuclear elements (LINE) accounted for 26.99%, 7.20%, and 2.99%, respectively. Both Gypsy (16.17%) and Copia (10.05%) LTRs were widely present in the *A. wilsonii* genome.

### Genome Evolution

To explore the evolutionary relationship between *A. wilsonii* and other plant species, we collected the data of 10 sequenced plant genomes, including genomes from five species of Sapindaceae (*Atalantia buxifolia, Citrus reticulate, Citrus sinensis, Citrus grandis*, and *Dimocarpus longan*) to construct a phylogenetic tree with 1,521 single copy ortholog genes shared from these genomes. (**Supplementary Table 13**). The resulting phylogenetic tree showed a sister relationship between *A. wilsonii* and *D. longan*, both of which are in the Sapindaceae. All of the Sapindaceae species were clustered into one monophyletic group. Using MCMCtree with calibrations, *A. wilsonii* and *D. longan* diverged from the last common ancestor at ~114.2 Mya, the divergence time between *A. wilsonii* and the most recent common ancestor of other Sapindaceae species at approximately 152.3 Mya (**Figure 2B**).

By comparing these genomes, a total of 18,267 orthologous genes were identified, including 2,090 genes unique to *A. wilsonii* (**Supplementary Figure 4**; **Supplementary Table 13**). In addition, evolution analysis of gene families revealed that 2,847 gene families in *A. wilsonii* expanded, whereas 771 families contracted (**Figure 2B**). Among the significantly expanded, or unique, gene families of *A. wilsonii*, functional annotation revealed that they were mainly enriched in Gene Ontology (GO) functional categories or Kyoto Encyclopedia of Genes and Genomes (KEGG) pathways, such as energy metabolism and transport, DNA reparation, glucose metabolism, hormonal signal transduction, biosynthesis of aromatic compounds, and flavonoid biosynthesis (**Supplementary Figures 5, 6**). Analysis of the *Ka*/*Ks* ratio of *A. wilsonii*, *D. longan*, *C. sinensis, A. buxifolia*, and *Vitis vinifera* also indicated that *A. wilsonii* had a moderate rate of gene evolution (**Supplementary Figure 7**).

### Whole-genome duplication of *A. wilsonii*

In order to explore whole-genome duplication events during *A. wilsonii* evolution, the age distribution of the duplication events was calculated by using the *Ks* values of the duplicated genes. Two main distribution peaks of *A. wilsonii* were found around the *Ks* values of 0.23 and 1.42 (**Figure 2C**). *Ks* 1.42 was consistent with the ancestral palaeohexaploidy event (γ event), which was shared by most core eudicots. The *Ks* 0.23 peak (∼17.69 Mya) suggested that a recent WGD event occurred in *A. wilsonii*. The same analysis with the genomes of *D. longan*, *C. sinensis, A. buxifolia*, and *V. vinifera* revealed that an occurrence of recent WGD event in *A. wilsonii* was lineage-specific and distinct from WGD events identified in other species (**Figure 2C**). Two *Ks* peak values at 0.3 and 0.64 were presented by comparing the homologous genes between *A. wilsonii* and *D. longan*, and between *A. wilsonii* and *C. sinensis*, respectively (**Figure 2C**). The result indicated that the recent WGD event in *A. wilsonii* occurred after the divergence of *A. wilsonii* from both *C. sinensis* and *D. longan.*

To confirm the recent WGD event presented in *A. wilsonii*, we performed a comparison of the syntenic depth ratio between *A. wilsonii* and the *V. vinifera* and *C. sinensis* genomes. We observed a similar pattern of syntenic depth ratio of four-to-two between *A. wilsonii* and *V. vinifera*, as well as between *A. wilsonii* and *C. sinensis* (**Supplementary Figure 8**). This indicates that a single syntenic region in both *V. vinifera* and *C. sinensis* is aligned to two *A. wilsonii* blocks (**Figure 2D**; **Supplementary Figure 9**). The results of the intergenomic co-linearity analysis provided clear support for the presence of a lineage-specific WGD event in *A. wilsonii*.

### Evolution of genes related to triterpenoid saponins

**Figure 3:**
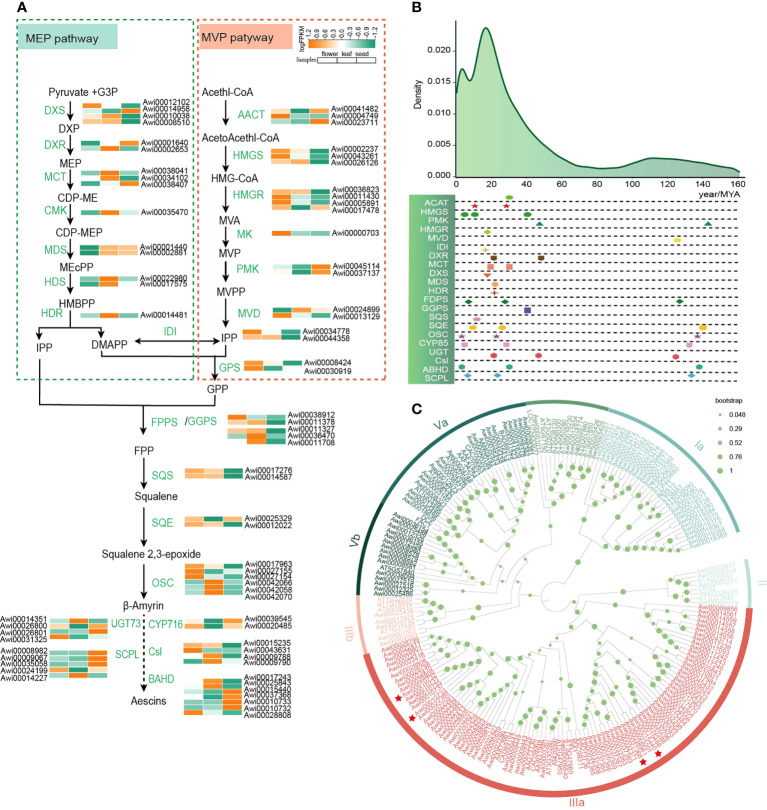
Evolution of genes involved in triterpenoid saponins in *A*. *wilsonii*. **(A)** Metabolic pathways and genes expression associated with biosynthesis and accumulation of triterpenoid saponins in *A. wilsonii* **(B)** The evolution of genes involved in triterpenoid saponins synthesis in *A*. *wilsonii*. **(C)** A phylogenetic tree of the BAHD genes in *A*. *wilsonii*. Among the seven branches, IIIA mostly uses acetyl-CoA as the main acyl donor, and accepts different alcohols as substrates to form esters. Genes marked with red stars were significantly expressed in seeds.

Aescins in *A. wilsonii* are the pentacyclic triterpene saponins of the oleanane type (Gruza et al., 2013), which are mainly produced through the mevalonate (MVA) and 2-c-methyl-d-erythritol-4-phosphate (MEP) metabolic pathways (**Figure 3A**). Isopentenyl diphosphate (IPP) is synthesized from pyruvate and glyceraldehyde-3-phosphate through seven enzyme reactions including DXS, DXR, MCT, CMK, MDS, HDS, HDR in plastids, and IPP is transported to the cytoplasm. In addition, acetyl CoA is the initial donor for producing dimethylallyl pyrophosphate (DMAPP) through the six enzymes AACT, HMGS, HMGR, MK, PMK, MVD in the cytoplasm. IPP and DMAPP can be converted to each other in the cytoplasm under the action of isopentenyl diphosphate-isomerase (IDI) (Pu et al., 2021) (**Supplementary Figure 10**). Under the catalysis of FPPS, GPPS, GPS, SQS, SQE, squalene 2,3-epoxide is formed from IPP and DMAPP as precursors (**Supplementary Figure 11**). The squalene 2,3-epoxide catalyzed by β-Amyrin Synthase (β-AS) in the OSC family to produce amyrin. The amyrin is further modified by triterpene-modifying (or tailoring) enzymes such as cytochrome P450s (CYP450), sugar transferases (UGTs), and acyltransferases (ACTs) to form a variety of triterpenoids (Thimmappa et al., 2014). According to the annotation and homology comparison with other species, the results show that genes in triterpene saponin pathway exist widely in the genome of *A. wilsonii* (**Supplementary Figure 12**).

The OSC family includes important enzymes involved in the formation of triterpenes. OSC catalyzes squalene 2,3-epoxide to form more than 100 triterpenes. β-AS is the only one that can catalyze the formation of oleanane type triterpenoid saponins (Xu et al., 2004). We identified 19 OSC genes. Three of the 19 OSC genes were β-ASs and 10 synthases with integrative functions (**Supplementary Figure13**). Genes encoding specific metabolic pathways are physically aggregated in plant genome genomes and often co induced (Nutzmann et al., 2016; Nutzmann et al., 2018). According to the research of OSC flanking region genes in different Brassicaceae, the core genes encoding triterpenes containing OSCs, CYP450s, and ACTs to form the dynamic gene clusters, diversified the enzymes to cope with evolutionary selection pressures (Liu et al., 2020a). In the *A. wilsonii* genome, we identified a total of 320 CYP450s (**Supplementary Figure 14**), and eight genes belonged to the CYP716 family and were reported to catalyze the C28 position in other plants (Nelson and Werck-Reichhart, 2011; Zhang et al., 2020). In addition, we identified 153 genes from the BAHD family (D'Auria, 2006; Johnson et al., 2011) and 46 genes from the serine carboxypeptidase-like (SCPL) acyltransferase family (Fraser et al., 2005). The members of BAHD family were divided into 7 classes, including Ia, Ib, II, IIIa, IIIb, Va, and Vb. The SCPL family was divided into the IA, IB, II, III, IV, V, VI classes (**Figure 3C**; **Supplementary Figure 15**). Among them, branch III in BAHD was reported to form esters with acetyl-CoA as the main acyl donor and various alcohols as substrates, and IA in the SCPL family has an acylation function.

We classified 230 members of the UGT family into 23 clans (**Supplementary Figure 16**). The glycosides of the aescin mainly consist of different trisaccharide chains from xylose, galactose, and glucose, and form glycoside bonds with C3-OH through glucuronic acid (Augustin et al., 2012). We identified 38 genes of the UGT73 family involved in glucuronic acid transfer in the genome of *A. wilsonii*. In addition, a total of 25 cellulose synthase-like genes were identified, of which 7 genes in clade Csl M were reported to enable glucuronic acid attach to the C-3 position during saponin synthesis (**Supplementary Figure 17**). The expression of these genes related to the triterpenoid saponins biosynthesis among various tissues showed that the genes related to terpenoid backbone biosynthesis (KO00900) and triterpenoid synthesis (KO00909) are abundantly expressed in *A. wilsonii* (**Figure 3A**). Especially in the late stage of saponin biosynthesis, the expression profiles showed that genes related to aescin biosynthesis such as *Awi00026800* and *Awi00026801* in the UGT73 family; *Awi00039545* and *Awi00020485* in CYP716; *Awi00015440*, *Awi00037368*, *Awi00010733*, and *Awi00010732* in the IIIa clade of the ABHD family; and *Awi00008982* and *Awi00009067* in the SCPL family were significantly expressed in seeds (**Figure 3A**).

By calculating the *Ks* for each duplicated gene pair associated with triterpenoid saponins synthesis, the major duplications of related genes in the synthesis pathway were generated in the recent WGD event (**Figure 3B**), suggesting that the occurrence of recent WGD event was significant for the evolution of triterpenoid saponins synthesis in buckeye.

### Population structure and species divergence

**Figure 4:**
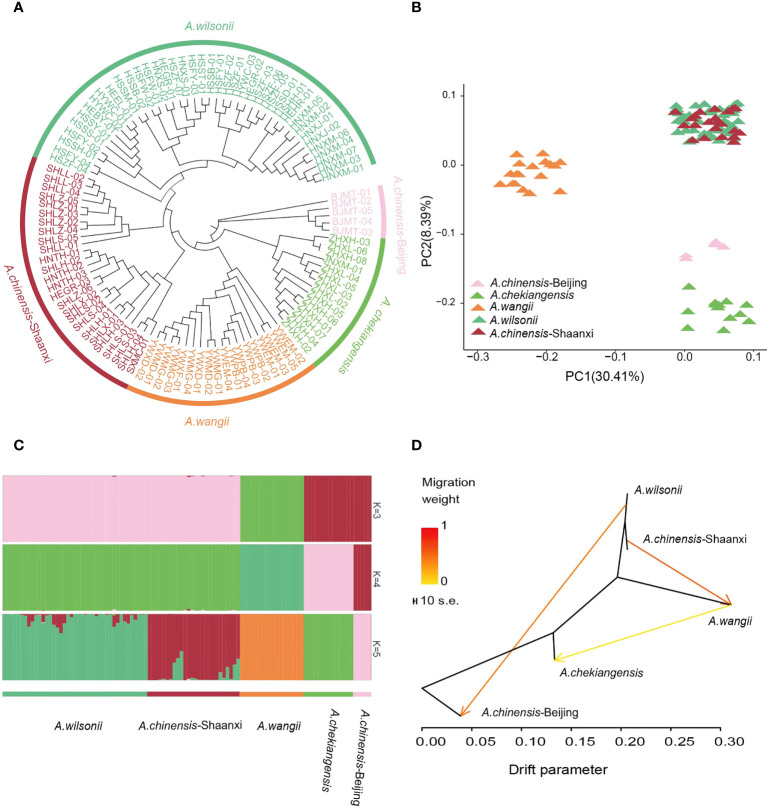
Population relationships and structures of buckeye. **(A)** Neighbor-joining phylogenetic tree of 104 buckeye accessions was constructed using SNPs. Colors represent different species. **(B)** PCA plots of 104 buckeye samples, with the first two principal 30.41% and 8.39%, respectively. **(C)** Model-based population assignment by ADMIXTURE analysis for K = 3-5. Each column represents a buckeye sample. **(D)** Gene-flow patterns that were detected among buckeyes groups using TreeMix. The shade of colors represented the weight of the migration events.

For understanding the population structure and divergence of the main *Aesculus* species in China, resequencing of 104 representative samples, including 41 A*. wilsonii*, 31 A*. chinensis*, 14 A*. chekiangensis*, and 18 A*. wangii* was performed (**Supplementary Table 1**). These samples were sequenced to an average depth of 5.27*×* and coverage of 94.08% of the *A. wilsonii* genome. The resulted in a total of 355.63 Gb of clean data available for population analysis (**Supplementary Table 1**). We identified 60,387,800 high-quality single nucleotide polymorphisms (SNPs). Genetic relationships between these samples were inferred using a neighbor-joining (NJ) tree on the basis of the identified SNPs. The tree revealed that all samples were clustered into five main groups. One group included *A. chekiangensis* samples collected from Zhejiang and Jiangsu provinces, one group included *A. wangii* samples from Yunnan province, and one group was mainly *A. wilsonii* samples from Hubei, Henan (**Figure 4A**). The *A. chinensis* samples were divided into two distinct groups. The samples from Beijing were close to the *A. chekiangensis* samples, while those from Shaanxi had a close relationship with the *A. wilsonii*.

The ADMIXTURE analysis and principal components analysis (PCA) also confirmed this pattern (**Figures 4B, C**). When *K* = 3 in the ADMIXTURE analysis, the *A. chinensis* Beijing and *A. chekiangensis* samples, as well as the *A. chinensis* Shaanxi and *A. wilsonii* samples, were grouped into two separate groups, and both were distinct from the samples of *A. wangii*. When *K* = 4, a subdivision between the *A. chinensis* Beijing and *A. chekiangensis* samples was seen. When *K* = 5, the *A. chinensis* Shaanxi samples further diverged from the *A. wilsonii* samples (**Figure 4C**; **Supplementary Figure 18**). Pairwise genome-wide fixation index (*F*_ST_) values between these *Aesculus* groups showed that genetic differentiation between *A. wangii* and other species groups (0.3166–0.5167) was significantly higher than other comparisons, such as those between *A. chekiangensis* and *A. wilsonii* (0.1805) and between group *A. wilsonii* and group *A. chinensis* Shaanxi (0.01300) (**Supplementary Table 14**). Variable levels of gene flow were observed between these specie groups (**Figure 4D**). Comparatively, *A. wilsonii* had the highest level of nucleotide diversity (π) (2.42 × 10^−3^) (**Supplementary Table 15**).

### Linkage disequilibrium and demography

**Figure 5:**
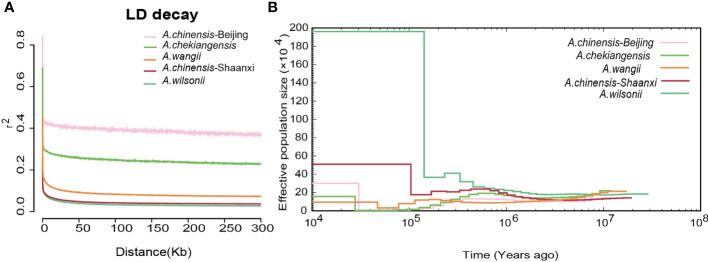
The Linkage disequilibrium and demography of buckeyes populations. **(A)** Patterns of different groups LD decay. **(B)** Demographic history of five buckeyes populations including *A*. *wilsonii*, *A*. *chinensis*-Shaanxi, *A*. *wangii*, *A*. *chekiangensis*, *A*. *chinensis*-Beijing.

Linkage disequilibrium (LD, measured as r2) decreased to half of its maximum values at 368 kb in *A. chinensis* but at 122 kb and 94 kb in *A. chekiangensis* and *A. wilsonii*, respectively (**Figure 5A**). The estimated LD values of species were inversely correlated with their population nucleotide diversity (**Supplementary Table 15**), as expected. To investigate the demographic history of different *Aesculus* species in China, pairwise sequentially Markovian coalescent (PSMC) analysis was used to estimate fluctuations in the effective population size (*N_e_*) from 20 million to 10,000 years ago. The changes in the *N_e_* of different species over time coincided with known times of climatic events. For each species investigated, the *N_e_* decreased in the early quaternary ice age (Ehlers and Gibbard, 2007) (∼2 Mya), and recovered during the last interglacial (∼140 kiloyears ago, kya) probably due to the rising temperatures (Petit et al., 1999). During the Last Glacial Maximum (LGM, ∼110 kya), *Aesculus* species populations contracted slightly, and the *N_e_* of *A. wilsonii* expanded rapidly from the end of LGM (**Figure 5B**).

## Discussion

### The *Aesculus wilsonii* Genome provides new resources for genetic diversity and functional studies of aesculus

Buckeye tree is known for its important medicinal and ornamental values. The seeds are rich in oil, flavonoids and saponins, and are widely used in bio-medicine and daily chemical industries. A reference genome is useful for analysis of the genetic background and secondary metabolic pathways of medicinal plants. Previous study reported that genome sizes of more than 10 species of *Aesculus* were previously predicted by flow cytometry (~467–623.48 Mb), and their chromosome numbers were also determined (2n = 2x = 40) (Krahulcová et al., 2017). Only the chloroplast genome of *A. wilsonii* (Liu et al., 2020b)and *A. wangii* (Zheng et al., 2017) had been reported, and no studies on the genome of buckeye tree have been reported worldwide. We present a high-quality chromosome-scale genome of *A. wilsonii* with the genome size of 579.01 Mb and estimated heterozygosity of 1.22%. A total of 94.72% of the assembled sequences were assigned to 20 pseudo-chromosomes with a scaffold N50 of 28.02 Mb. Compared with other species of Sapindaceae, the genome size of *A. wilsonii* is close to the genome size of longan (455.5 Mb, 2n=30) (Wang et al., 2022), litchi (470 Mb, 2n=30) (Hu et al., 2022), *Sapindus mukorossi* (432.29 Mb, 2n=28) (Xue et al., 2022), and *Acer truncatum* (628.84 Mb, 2n=26) (Ma et al., 2020) with a diploid genome, although the chromosome number of *A. wilsonii* is greater than them. On the other hand, we identified 60,387,800 high-quality SNPs by resequencing of 104 wild *Aesculus* samples from China, and clarified the genetic relationships between *A. wilsonii* and *A. chinensis*, and *A. chekiangensis* and *A. wangii*. These rich genomic resources for future molecular breeding and biological studies of *Aesculus*.

### The WGD events were vital for *A. wilsonii* addressing environmental challenges and functional diversity

Polyploidy events are common in plants and have played an important role in plant evolution and adaptation (Wu et al., 2020). Compared with other species such longan, litchi, *Sapindus mukorossi*, *Citrus sinensis*, *Acer truncatum* which had only one ancient γ-WGD event, a recent specific WGD event identified in *A. wilsonii* (**Figure 2C**) was unexpected. The recent WGD event in *A. wilsonii* occurred at about 17.69 Mya, during the Oligocene Ice Age when the climate changed dramatically, and also when many other plant WGD events occurred (Blanc, 2004). Environmental factors such as a cold climate, climate change due to asteroid impact, and darkness are all potential drivers of plant polyploidy (Wu et al., 2020). As a result of plants responding to environmental and climatic changes, duplications of their whole genomes can rapidly increase genomic contents and genetic variation. This can provide adaptive advantages through species biased gene expression contributing to novel gene regulation networks and signal transductions (Freeling, 2009). The results of specific and expanded genes of *A. wilsonii* are related to light and oxygen reaction, biosynthesis of secondary metabolites, such as terpenoids, flavonoids, plant pathogen interaction, and DNA repair genes, further providing support for this conclusion. The survival probability of *A. wilsonii* under environmental changes may therefore benefit from the WGD event.

WGDs also contribute to the innovation of species-specific traits such as specific metabolites (Liu et al., 2021). Genomic studies of triptolide (Tu et al., 2020) and lavender (Li et al., 2021) showed that genes related to the terpenoid biosynthesis pathway were also duplicated during the WGD events. The duplication of some gene families caused by WGDs led to the diversity of terpenoids. In particular, the expansion of TPS, CYP450, and BAHD families greatly enriched the variety of terpenoids. Aescins are the only pentacyclic triterpenoid saponins existing in *Aesculus*. We found that the gene families related to the biosynthetic pathway of terpenoid metabolism in the genome of *A. wilsonii* were duplicated in different periods (**Figure 3B**). This was especially true for the gene families involved in the late stage of terpene synthesis such as CYP450, UGT, BAHD, and SCPL, greatly enriching the different species of pentacyclic triterpenoids.

### Preliminary identification of genes specific to the biosynthesis of aescins in *A. wilsonii* genome

Aescins are the main characteristic active components of Aesculus species. Due to its important pharmacological activities such as anti-inflammatory, anti-edema, and inhibition of cancer cell proliferation, various pharmaceutical preparations containing aescins extract have been used in clinical treatment. Based on the *A. wilsonii* genome, the metabolic pathway of aescin biosynthesis was preliminarily analyzed. Analysis of unique genes in *A. wilsonii* genome showed that a significant enrichment of genes related to terpene biosynthesis metabolic pathways such as HMGR, MCT, GPPS, SQE, BAHD. Structural and activity analysis showed that the acyl groups on C-21 and C-22 in these saponins play an important role in their activity(Zhizhen Zhang et al., 2010). We identified *Awi00015440, Awi00037368* genes in the unique genes, which belong to the IIIa subfamily of BAHD with acylation, and significantly expressed in seeds (**Figures 3A**, **C**). In other plants, homologous genes have been shown to have acylated functions (D'Auria, 2006).

### Reclassification of buckeye in major regions of china and the bottleneck in the evolutionary of some population

Population genomics analysis revealed the clear genetic relationship of *Aesculus* species in China except for *A. chinens*is (**Figure 4A**). The *Aesculus* plants naturally occurring in Beijing and Shaanxi were traditionally identified as *A. chinensis* (FANG, 1981). However, our results showed that the samples of *A. chinensis* collected from Beijing and southern Shaanxi were closer to those of *A. chekiangensis* and *A. wilsonii*, respectively (**Figures 4B, C**). Given the relatively contiguous or overlapping geographical distributions of *A. wilsonii* (mainly in western Hubei and southwest Henan) and *A. chinensis* (from southern Shaanxi), these samples may belong to the *A. wilsonii* species. The slight morphological differences between them may be due to the influence of niche differentiation (Du et al., 2020). The close relationship between the *A. chinensis* Beijing and the *A. chekiangensis* samples is consistent with the record that *A. chekiangensis* is a variety of *A. chinensis* (FANG, 1981). Compared to *A. wangii* from Yunnan, the overall genetic differentiation between *A. wilsonii*, *A. chinensis*, and *A. chekiangensis* is weaker (**Supplementary Table 14**). In the Pharmacopoeia of the People’s republic of China (2020 edition), the seeds of *A. wilsonii*, *A. chinensis*, and *A. chekiangensis* are all used as TCM of Semen Aesculi. The close relationship between them may reflect a similar genomic basis, which in turn can improve the generation of consistent quality Semen Aesculi and thus benefit human health.

The *A. wilsonii* populations from the Qinling Mountains had a higher nucleotide polymorphism and faster LD decay than the other species populations (**Figure 5A**). This result was consistent with the demographic analysis, in which the range of *A. wilsonii* expanded significantly in the recent past, while the population expansions of *A. chinensis, A. chekiangensis*, and *A. wangii* were much less obvious (**Figure 5B**). In the process of species evolution, the genetic diversity of populations is affected by many factors such as the reproduction system, genetic drift, natural selection, gene flow, and human disturbance (Nevo, 2001; Frankham R and Brisoe, 2002). Among the four *Aesculus* species studied here, *A. wilsonii* has a relatively extensive natural distribution and its populations have been further increased by widespread artificial plantings in the Qinling mountain area. This is because its medicinal and ornamental values have long been recognized by local people. In contrast to *A. wilsonii*, the natural distribution of *A. chinensis*, *A. chekiangensis*, and *A. wangii* is restricted and limited utilization of their seeds has slowed down their population expansion. In addition, limited pollen transmission distance and large seeds can also affect population growth and range expansion (Isagi et al., 2007), and can lead to the population of *A. chinensis*, *A. chekiangensis*, and *A. wangii* experiences population bottlenecks during evolution.

## Conclusion

In conclusion, lineage-specific WGD events and the related candidate genes involved in the biosynthesis of triterpenoid saponins were identified by assembling to the chromosomal level high-quality *A. wilsonii* genome. Population resequencing of four *Aesculus* species in China further clarified their genetic and evolutionary relationships. In the future, we will use recombinant proteins expressed in *E. coli* and gene editing techniques to functionally characterize the aescins biosynthetic genes. In addition, the small size of the present study population has limited genetic diversity of *Aesculus*. The more samples will be collected for gene sequencing and phenotype collection analysis to further investigate their relationship and identify genes of excellent quality. These studies provide insights that should be significant for the conservation and utilization of *Aesculus*.

## Data availability statement

The datasets presented in this study can be found in online repositories. The names of the repository/repositories and accession number(s) can be found below: https://ngdc.cncb.ac.cn/, CRA007235, https://ngdc.cncb.ac.cn/, CRA007228.

## Author contributions

LY, YL, SC, and ZS conceived and supervised the project; LY, ZS collected the samples; LLY and GC performed the raw data analysis; LY and LJ analyzed the gene families; LY and YL wrote and revised the paper; BW, ZH contributed substantially to the revisions. All authors contributed to the article and approved the submitted version.

## Funding

This work was supported by the National Key Research & Development Program of China (2017YFC1701000 and 2019YFC1711100).

## Conflict of interest

Author LY and GC were employed by the company Wuhan Benagen Tech Solutions Company Limited.

The remaining authors declare that the research was conducted in the absence of any commercial or financial relationships that could be construed as a potential conflict of interest.

## Publisher’s note

All claims expressed in this article are solely those of the authors and do not necessarily represent those of their affiliated organizations, or those of the publisher, the editors and the reviewers. Any product that may be evaluated in this article, or claim that may be made by its manufacturer, is not guaranteed or endorsed by the publisher.
