# Supplementary material for: The Chromosome-level genome of Aesculus wilsonii provides new insights into terpenoid biosynthesis and Aesculus evolution
Source: Front Plant Sci. 2022 Oct 18;13:1022169. doi: 10.3389/fpls.2022.1022169 (PMC9642078; doi:10.3389/fpls.2022.1022169)
Supplement: Supplementary file 1 [file DataSheet_1.docx]

**
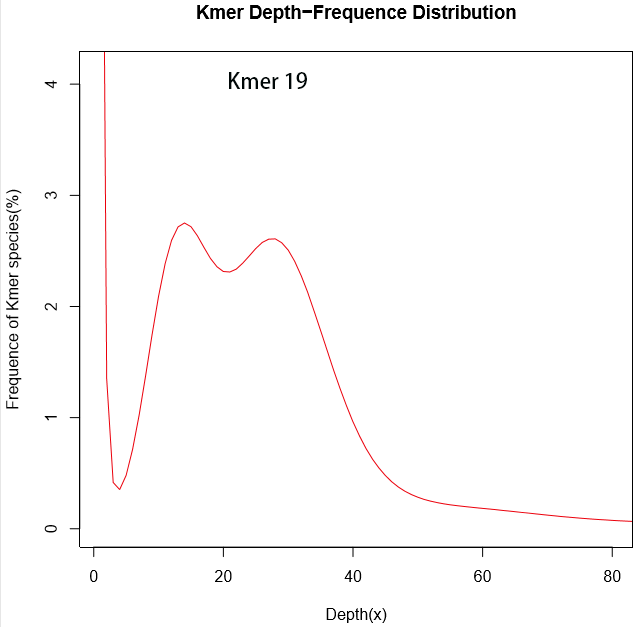
**

**Supplementary Figure1.** K-mer analysis for the estimation of genome size and heterozygosity of *A. wilsonii*.

**
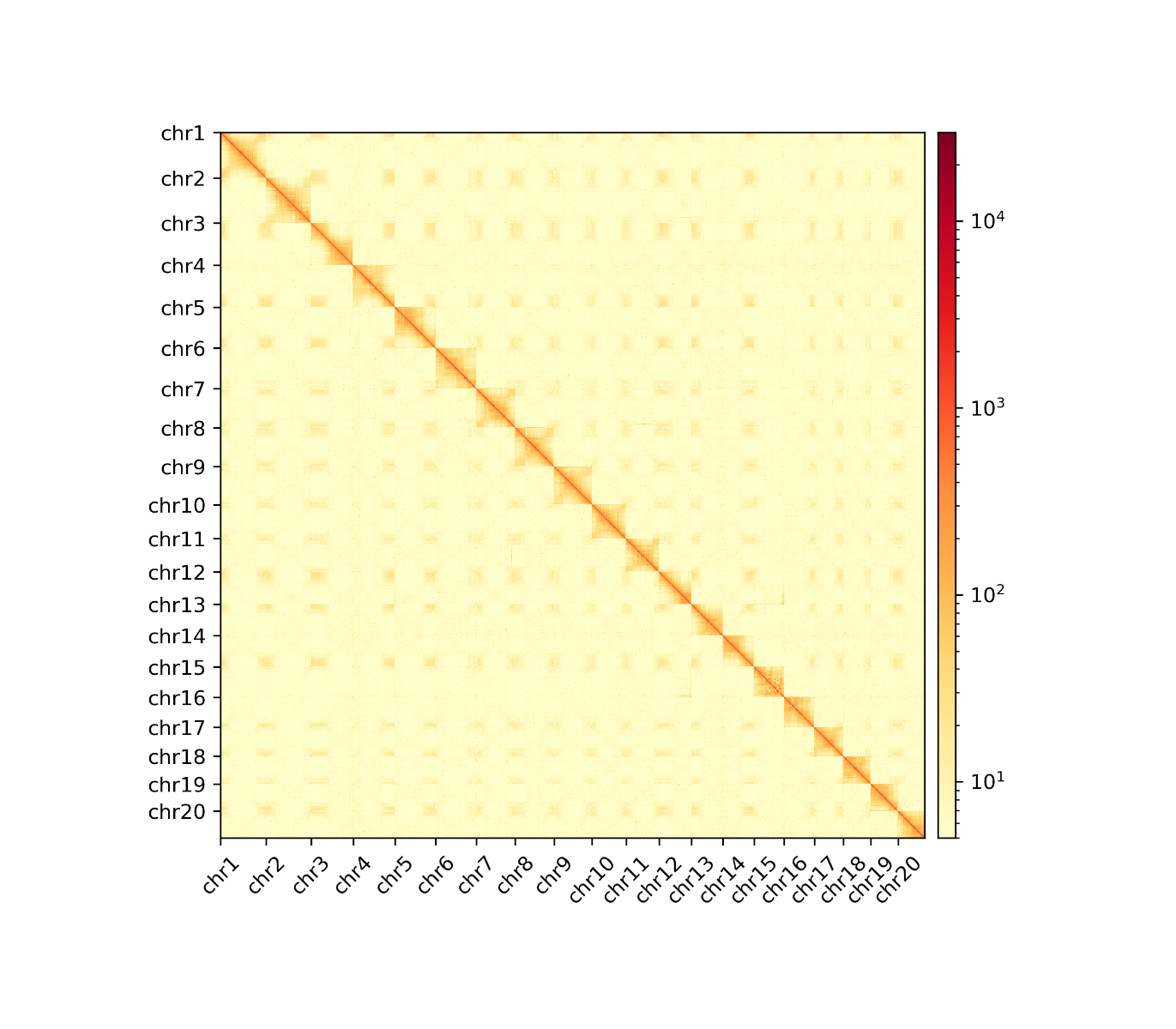
****Supplementary Figure2.** Intensity signal heat map of Hi-C chromosome interaction.


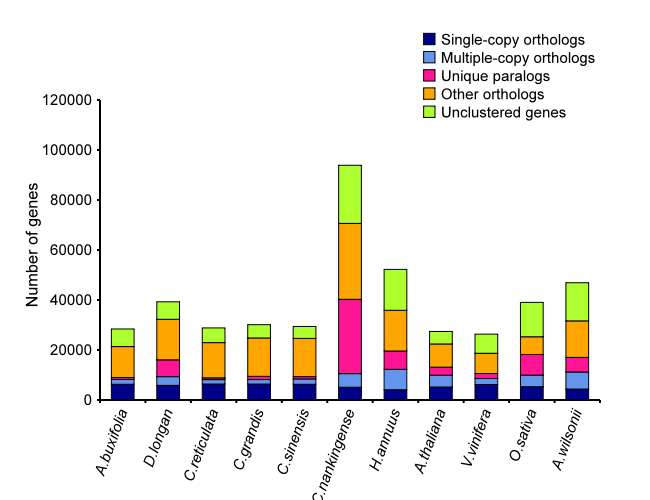


**Supplementary Figure3.** Orthologous genes in A. wilsonii and other 10 plant genomes

**
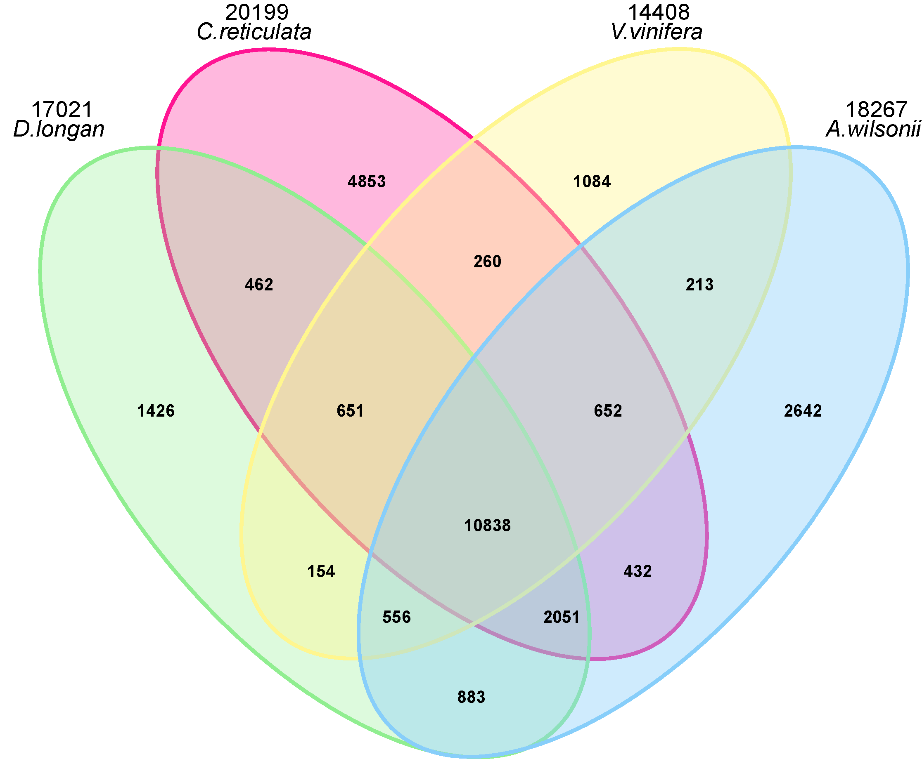
**

**Supplementary Figure4.** Venn diagram shows the number of orthologous genes families in *A. wilsonii* genome with *D. longan*, *C. reticulata*, *V. vinifera*.


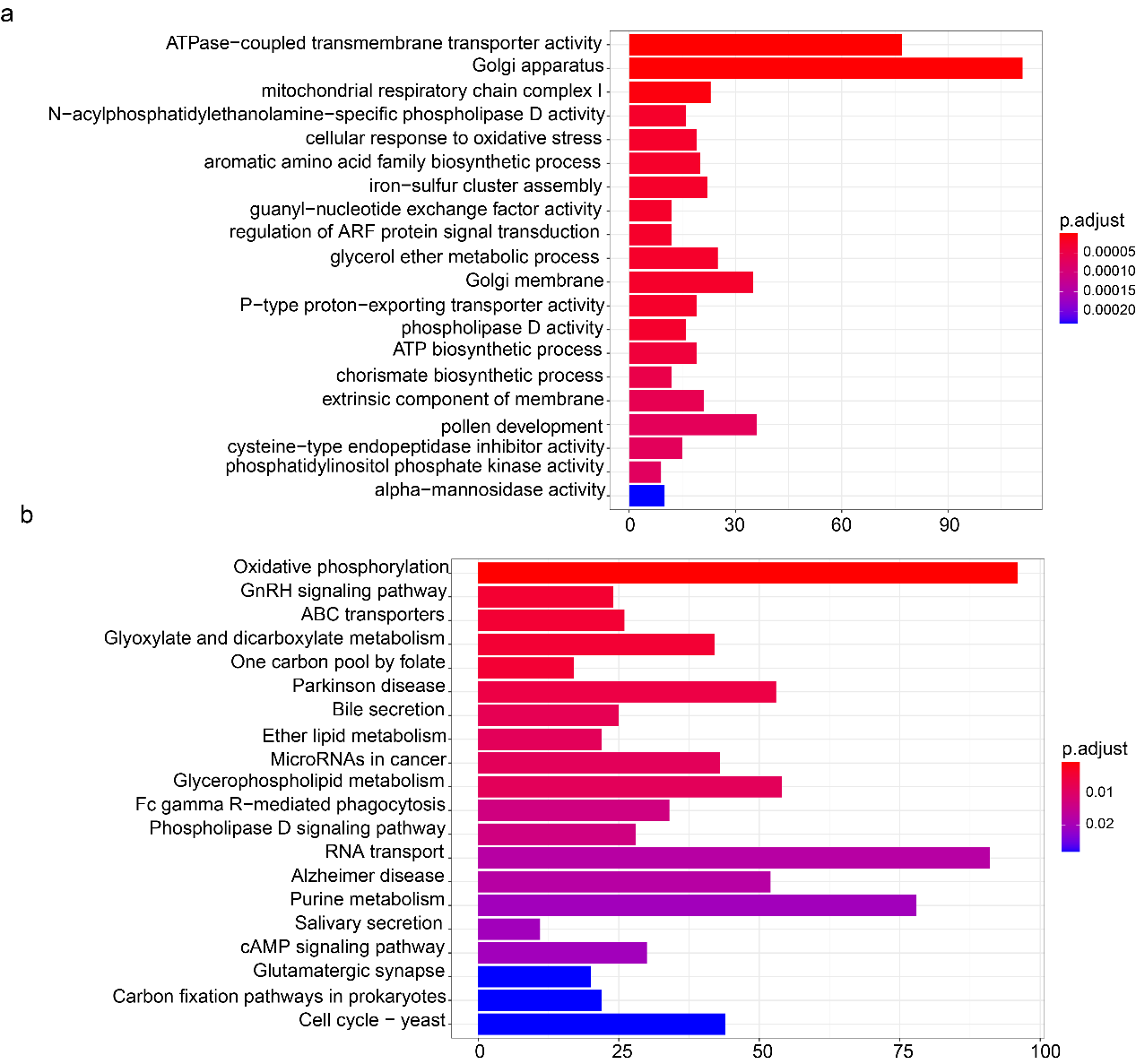


**Supplementary Figure5.** GO term (a) and KEGG pathway(b) enrichment of expansion genes in the *A. wilsonii* genome.


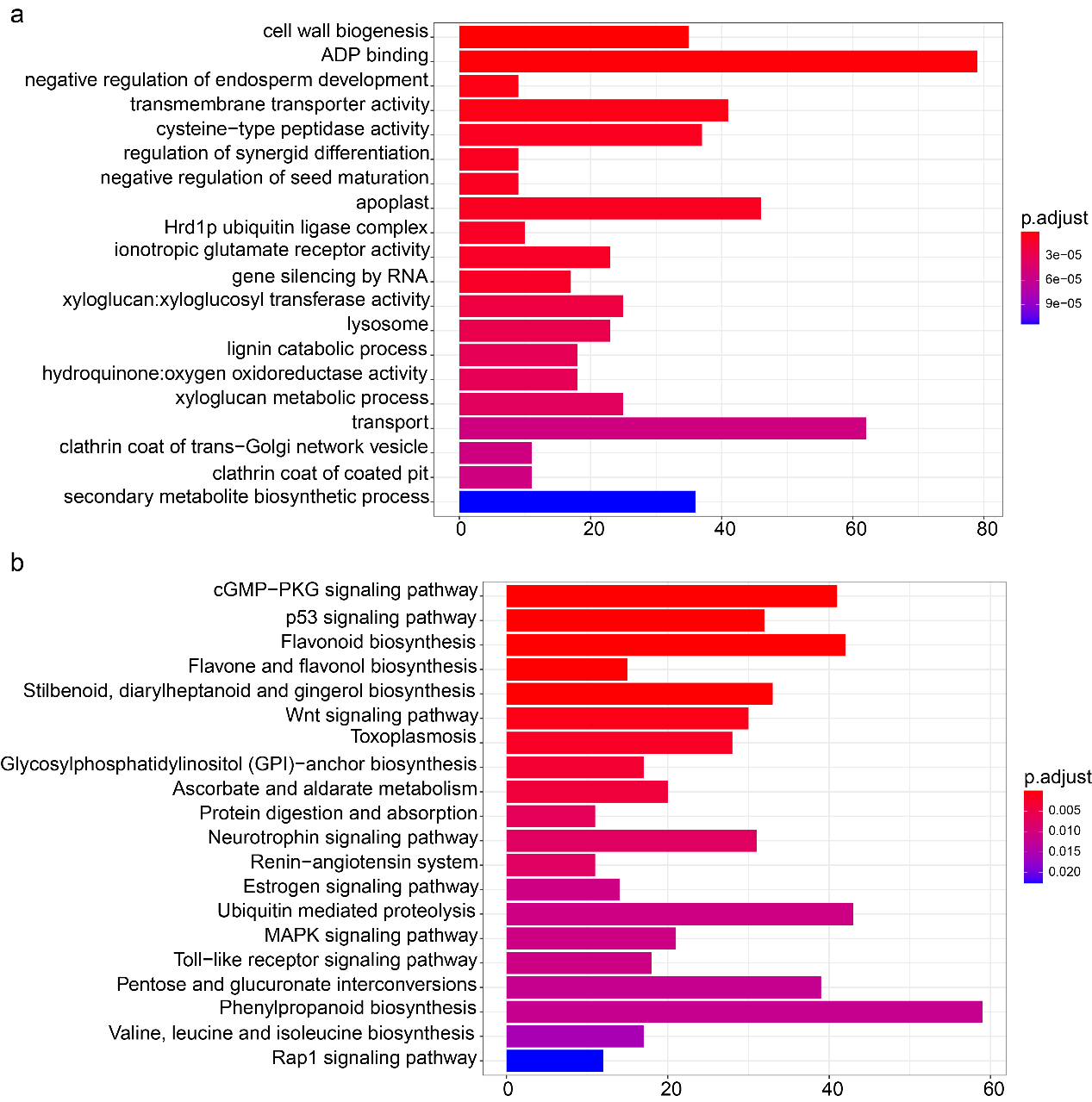


**Supplementary Figure6.** GO term (a) and KEGG pathway(b) enrichment of unique genes in the *A. wilsonii* genome.

**
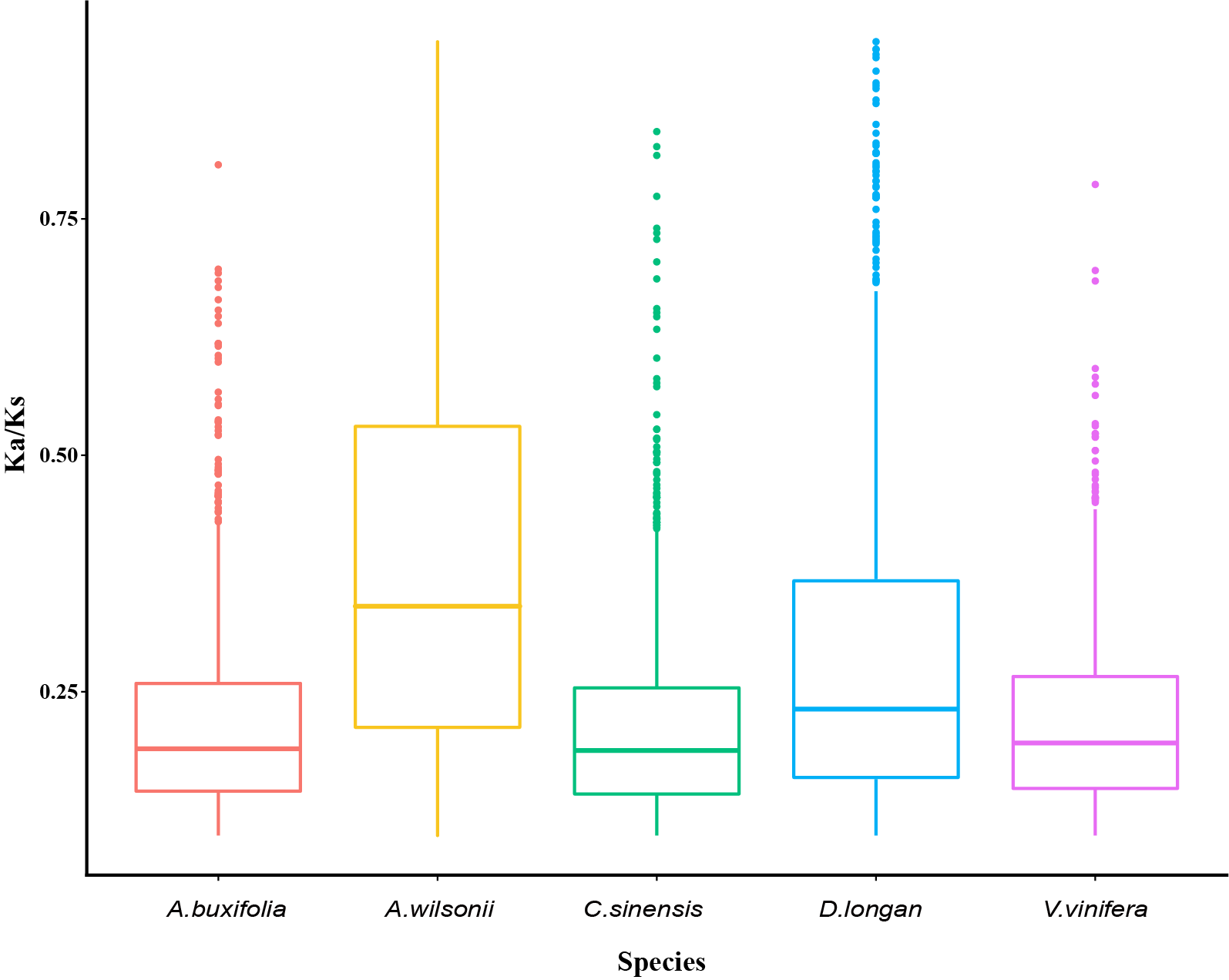
**

**Supplementary Figure7.** Ka/Ks ratio between different species.


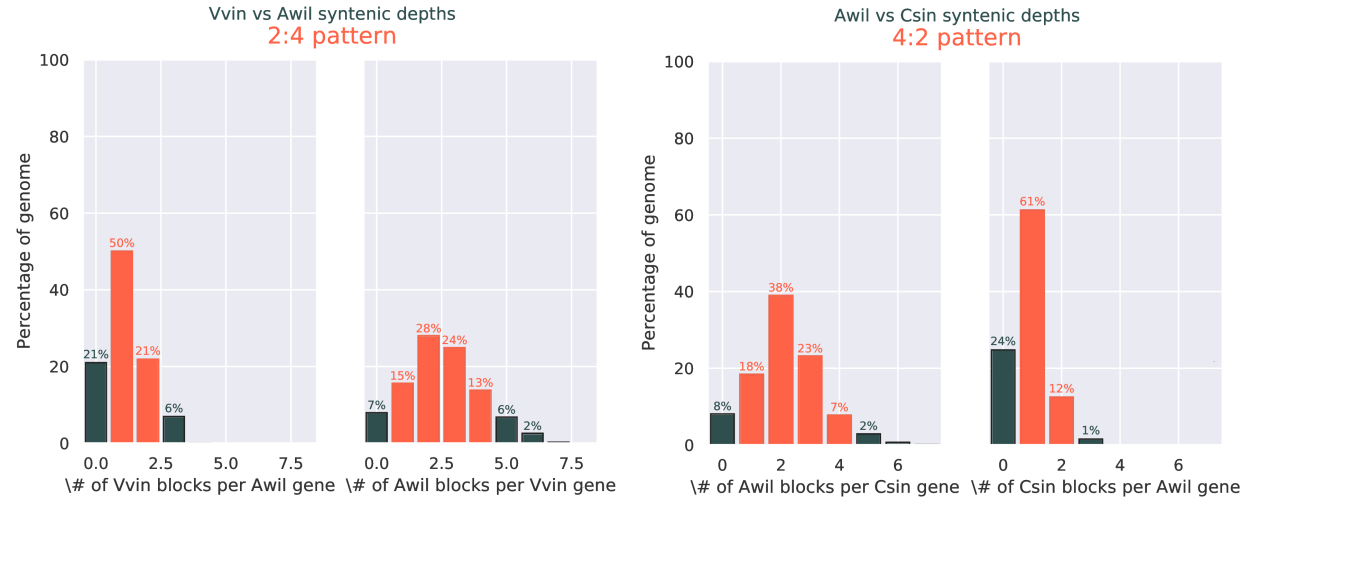


**Supplementary Figure****8.** The syntenic depth ratio between *A. wilsonii* (Awil) and *V. vinifera* (Vvin), *C. sinensis* (Csin).


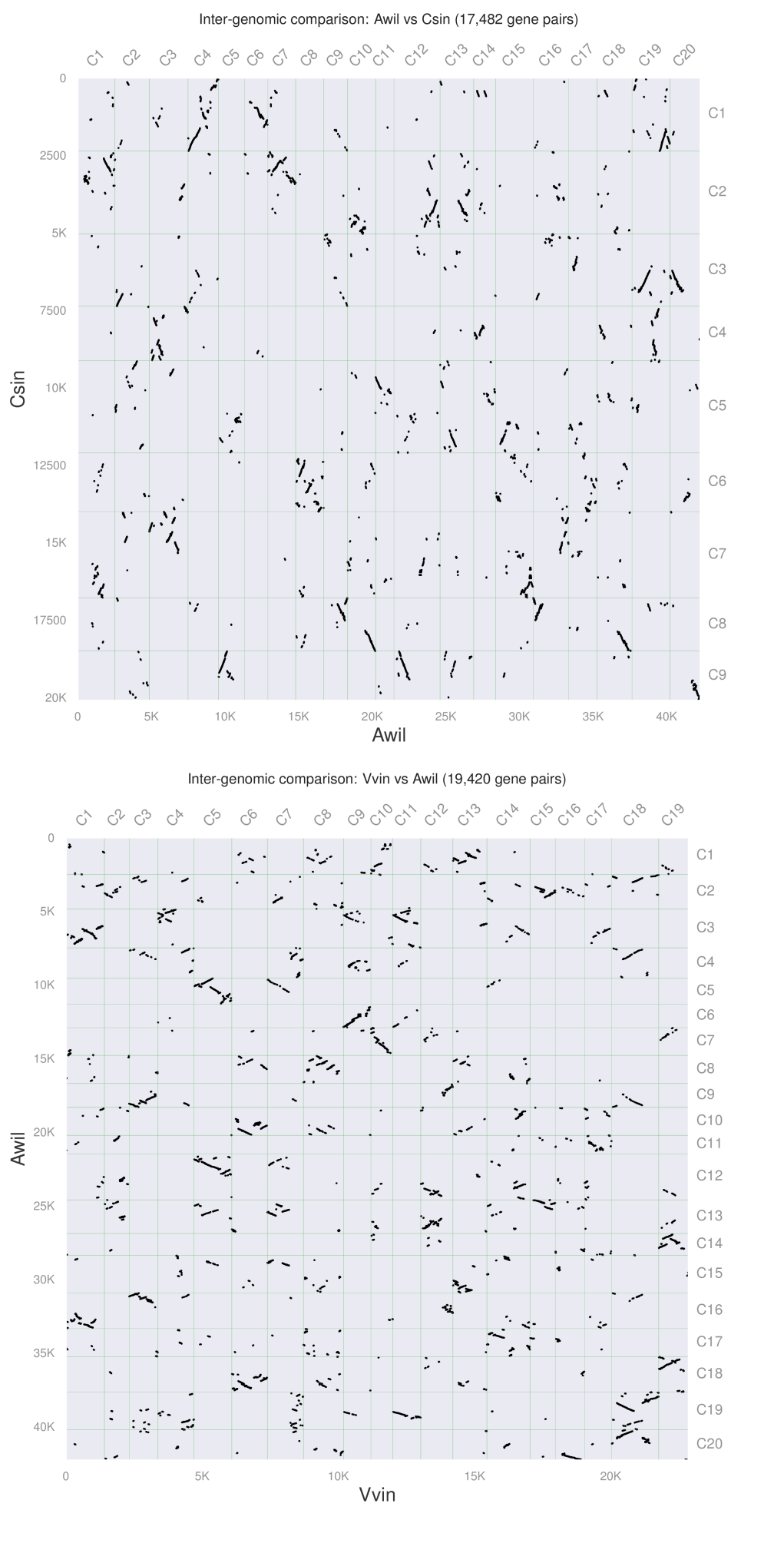


**Supplementary Figure9.** Syntenic dotplot illustrating the comparative analysis between *A. wilsonii* (Awil) and *V. vinifera* (Vvin), *C. sinensis* (Csin) *g*enomes. C1-C20 are twenty chromosome-scale pseudomolecules of *A. wilsonii* genome assembly.


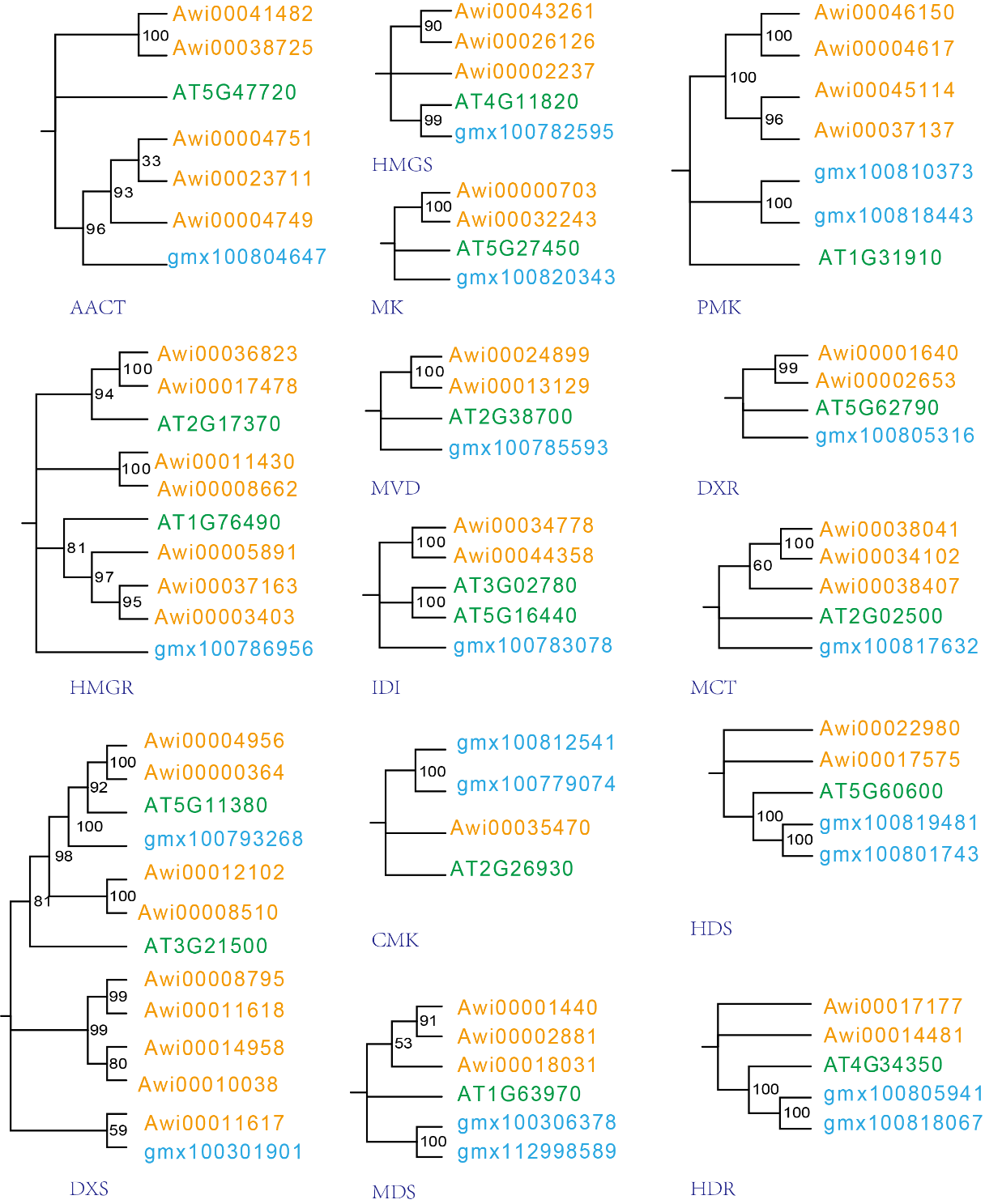


**Supplementary Figure10.** **Phylogenetic analysis of terpenoid backbone biosynthesis related gene families in MVA and MEP pathways.**

Green and blue colors correspond to *Arabidopsis thaliana* and *Glycine max*，the order of gene family name as follow: acyl-coenzyme A-cholesterol acyltransferase (AACT), hydroxymethylglutaryl coenzyme A synthase (HMGS), hydroxymethylglutaryl coenzyme A reductase (HMGR), mevalonate kinase (MK),phospho-mevalonate kinase (PMK), mevalonate diphosphate decarboxylase (MVD), isopentenyl diphosphate isomerase (IDI), 1-deoxy-D-xylulose 5-phosphate synthase (DXS), 1-deoxy-D-xylulose 5- phosphate reductoisomerase (DXR), 2-C-methyl-Derythritol-4-phosphate cytidylyltransferase (MCT), 4-(cytidine-5-diphospho) -2-C-

methyl-D-erythritol kinase (CMK), 2-C-methyl-D-erythritol-2,4-cyclodiphosphate synthase (MDS), (E)-4-hydroxy-3-methyl-but-2-enyl-pyrophosphate synthase (HDS), (E)-4-hydroxy-3-

methyl-but-2-enyl-pyrophosphate reductase (HDR).


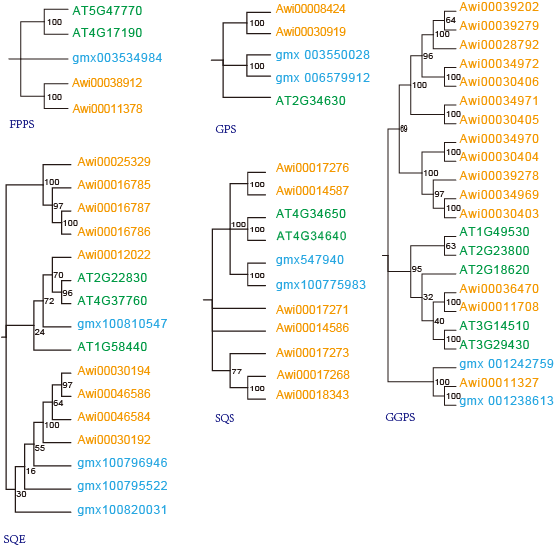


**Supplementary Figure****11. Phylogenetic analysis of triterpenoid skeleton biosynthesis-related gene families.** The green and blue colors correspond to *Arabidopsis thaliana* and *Glycine max*. Different colors correspond to different species and the order of gene family name as follow: farnesyl pyrophosphate synthase (FPPS), geranyl diphosphate synthase (GPS), geranylgeranyl diphosphate synthase (GGPS), squalene epoxidase (SQE), squalene synthase (SQS).


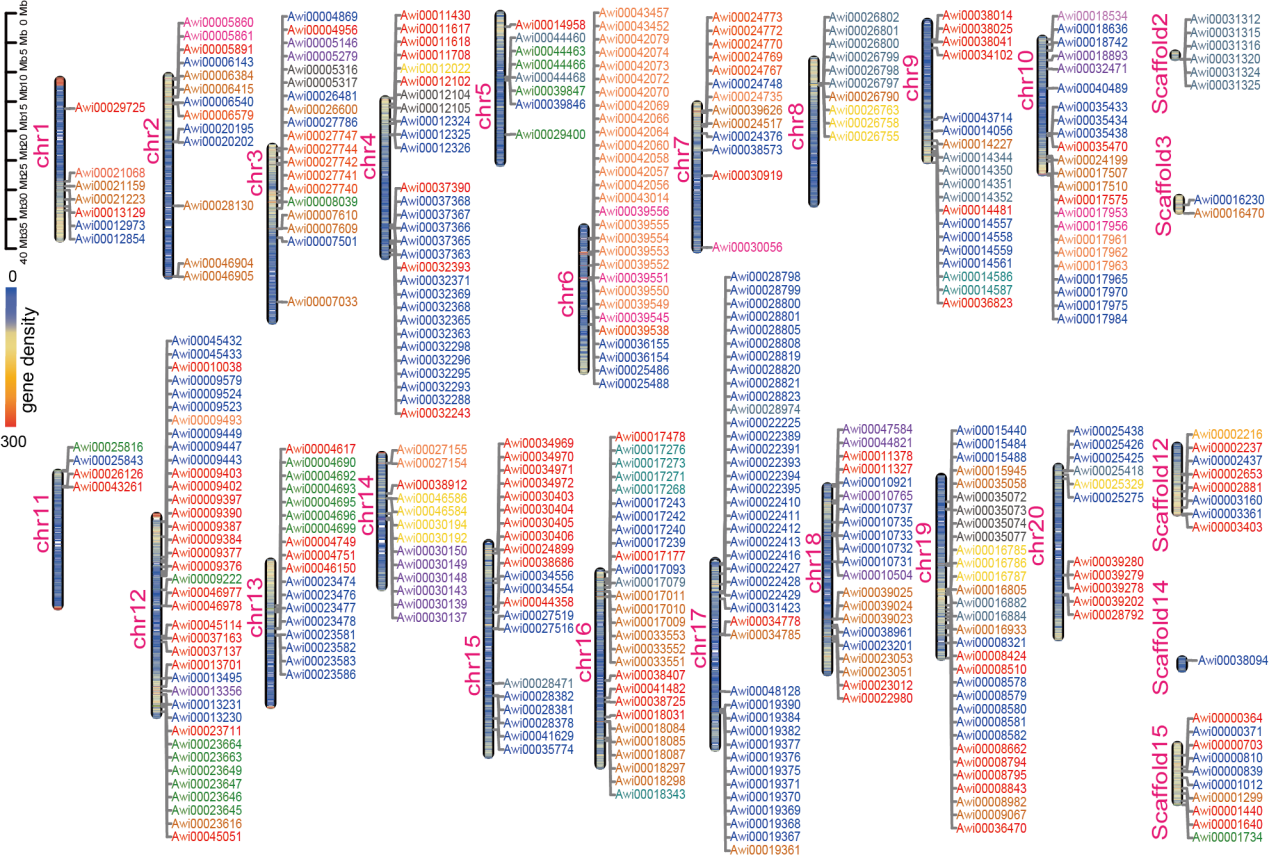


**Supplementary Figure 12. Chromosome location of triterpenoid saponins biosynthesis genes**. Genes colored with red, cyan 4, gold 1, sienna 1, chocolate, blue, tomato, deep pink, purple, orange1, orchid, orange red, forest green, skyblue4, grey31 indicate genes involved in MEP/MVA pathway, SQS, SQE, OSC, SCPL, ABHD, CYP51, CYP716, CYP72, CYP87, CYP93, CYP71, UGT74, UGT73 and UGT91 gene families, respectively. The color depth on each chromosome represented gene density per 1000000bp.


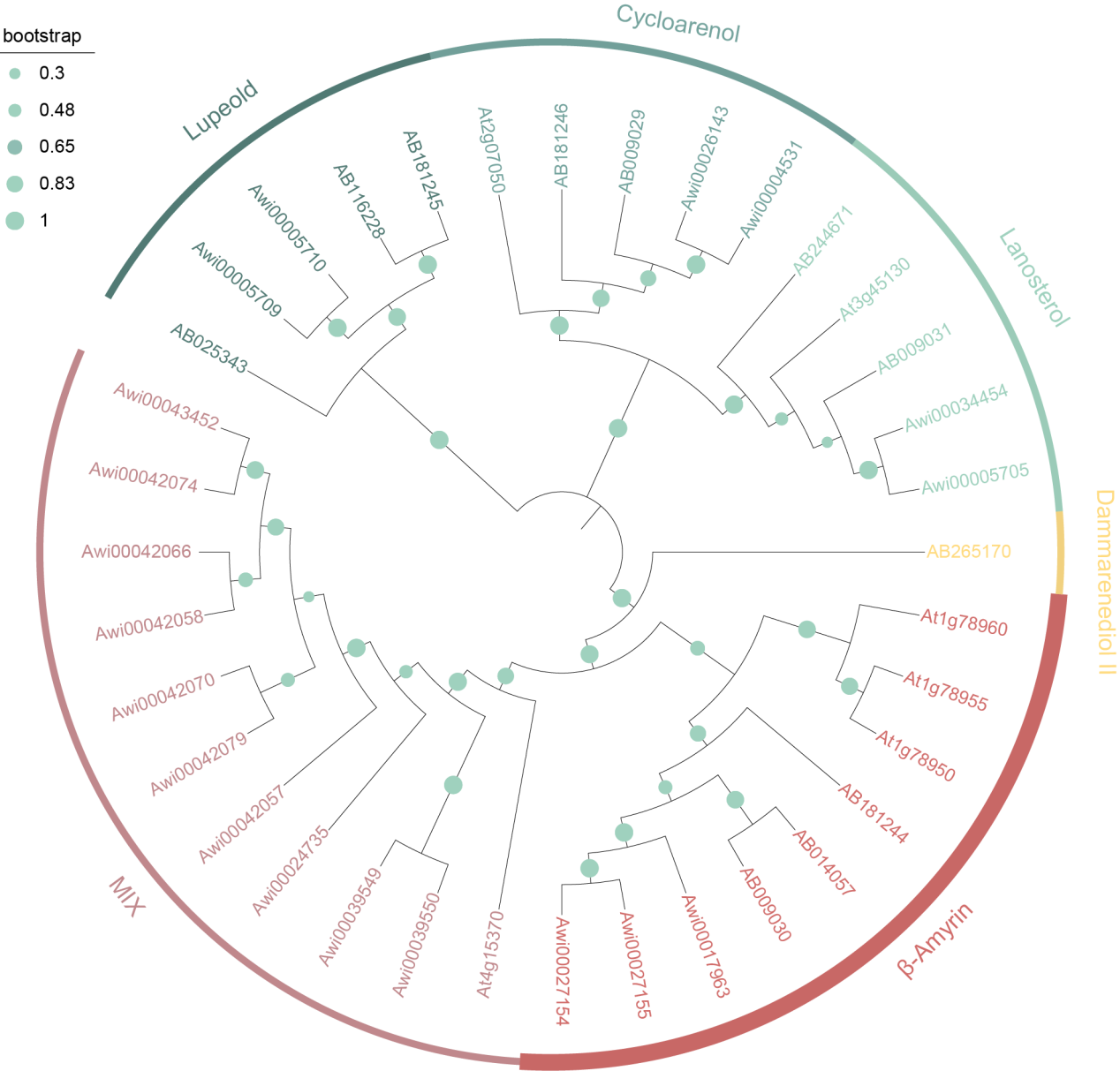


**Supplementary Figure13.** A phylogenetic tree of the OSC (oxidized squalene cyclase) genes in *A. wilsonii*.


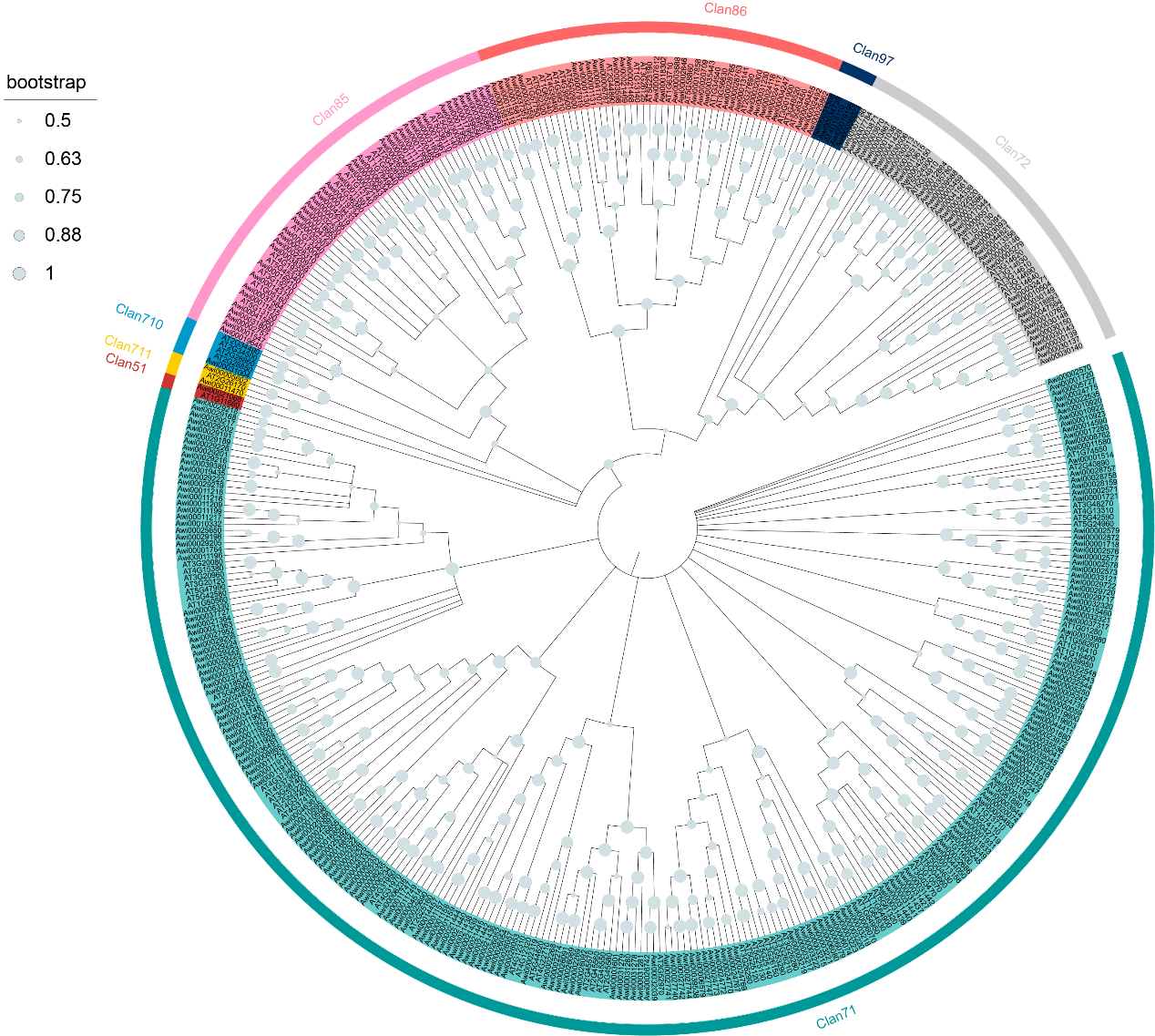


**Supplementary Figure14.** A phylogenetic tree of the CYP450 genes in *A. wilsonii*. The Neighbor-Joining phylogenetic tree was constructed by the total CYP450 members of *A. wilsonii and* Arabidopsis*.* The grayish circles indicated the bootstrap values greater than 50. Branches colored with green, red, yellow, azure, pink, flesh pink, dark blue, gray indicate members of CYP71, CYP51, CYP711, CYP710, CYP85, CYP86, CYP97 and CYP72 clan, respectively.

**
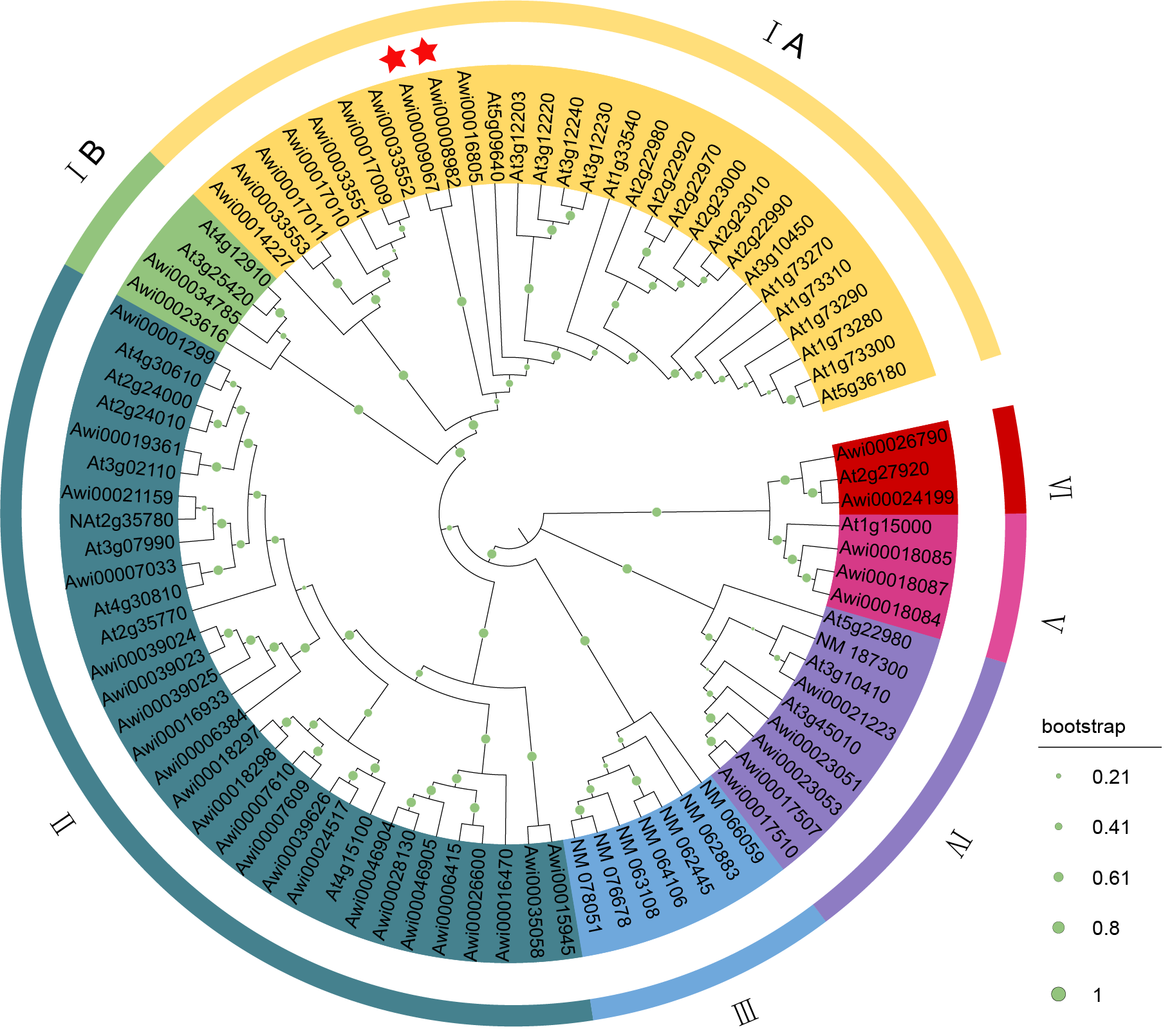
**

**Supplementary Figure15.** A phylogenetic tree of the SCLP genes in *A. wilsonii*. The Neighbor-Joining phylogenetic tree was constructed by the total SCLP members of *A. wilsonii* with Arabidopsis. Branches symbolized with red, magenta, purple, blue grey, green, light green, light yellow indicate members ofⅠA,ⅠB,Ⅱ,Ⅲ,Ⅳ,Ⅴ,Ⅵ.The genes that had been identified in clade IA have acyltransferase function, genes marked with red stars were highly expressed in seeds.


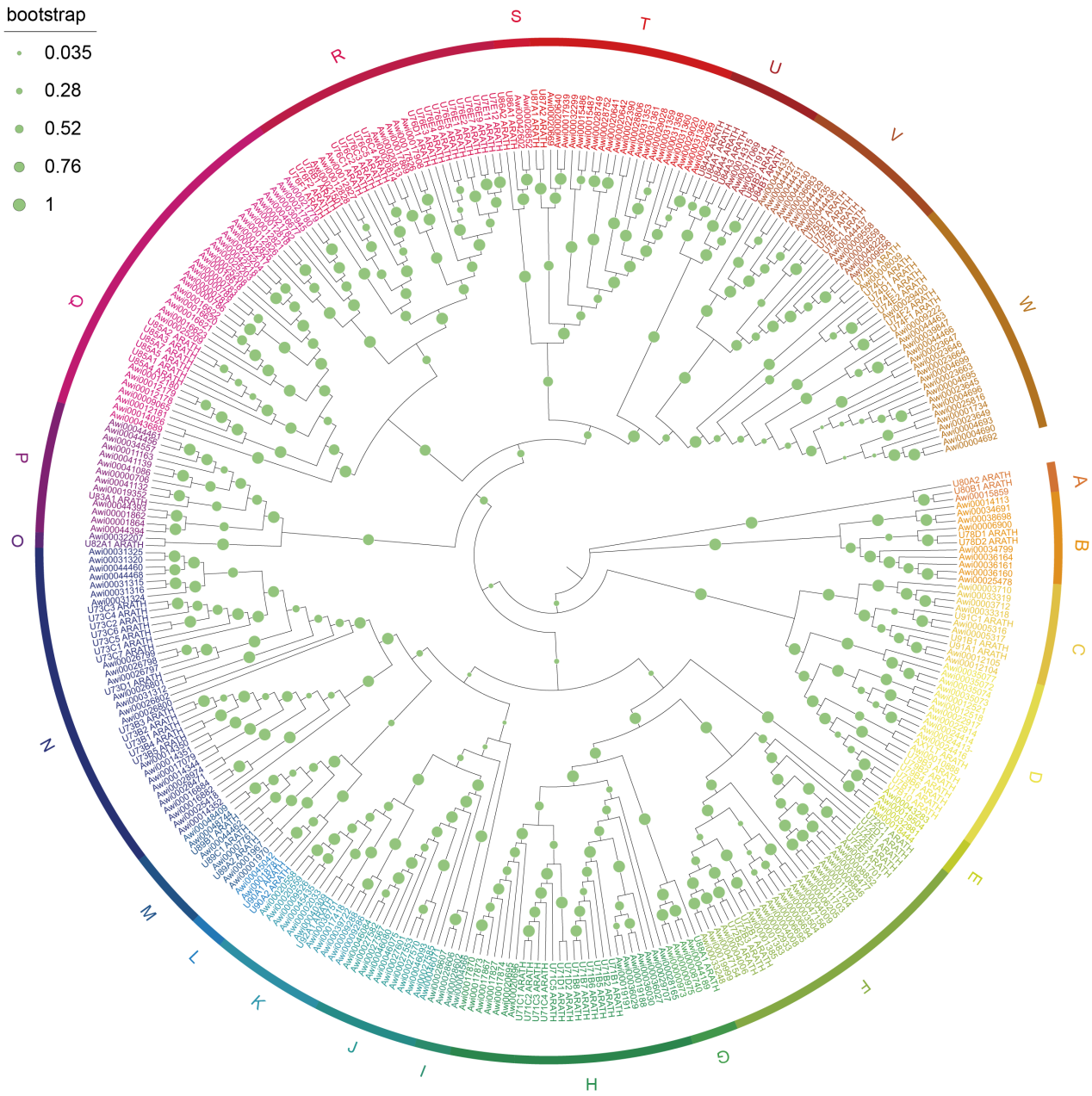


**Supplementary Figure16. A phylogenetic tree of the UGT genes in *A. wilsonii*.** The Neighbor-Joining phylogenetic tree was constructed by the total UGT members of *A. wilsonii* with Arabidopsis*.* The grayish circles indicated the bootstrap values greater than 50. Branches symbolized with the A, B, C, D, E, F, G, H, I, J, K, L, M, N, O, P, Q, R, S, T, U, V, W, X indicate members of UGT80,UGT78,UGT91,UGT79, UGT708,UGT72,UGT88, UGT 71, zeatin O-glucosyltransferase-like, flavanone-O-

beta-L-rhamnosyltransferase-like, UGT 92, UGT 90, UGT 89, UGT 73, UGT 82, UGT 83, UGT 85, UGT 76, UGT 86, UGT 87, UGT 84, UGT 75, UGT 74.

**
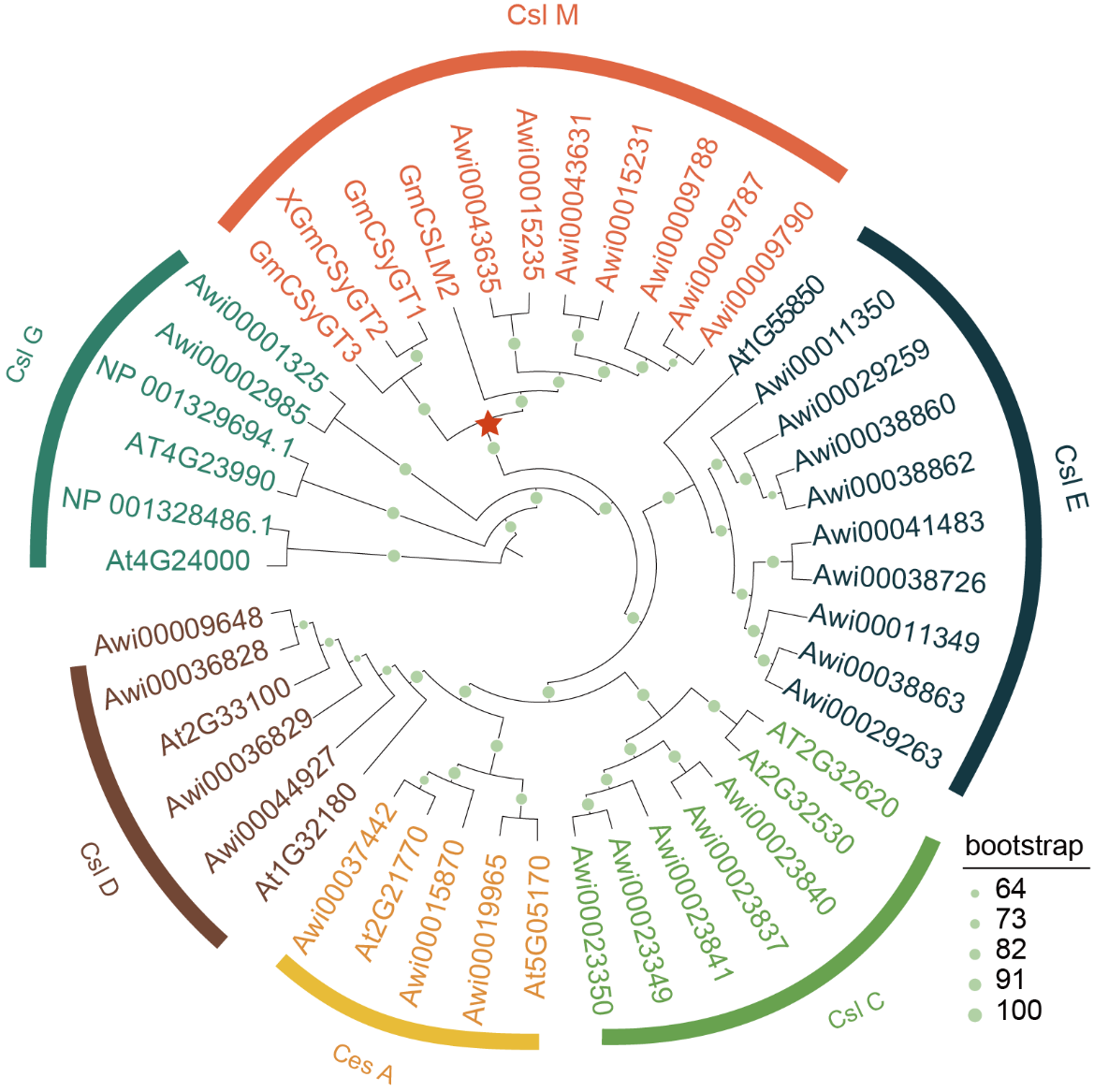
**

**Supplementary Figure17.** Phylogeny of cellulose synthase-like (Csl) families. Genes in Csl clade with star was associated with catalysis of 3-O-glucuronosylation of triterpenoid aglycones.


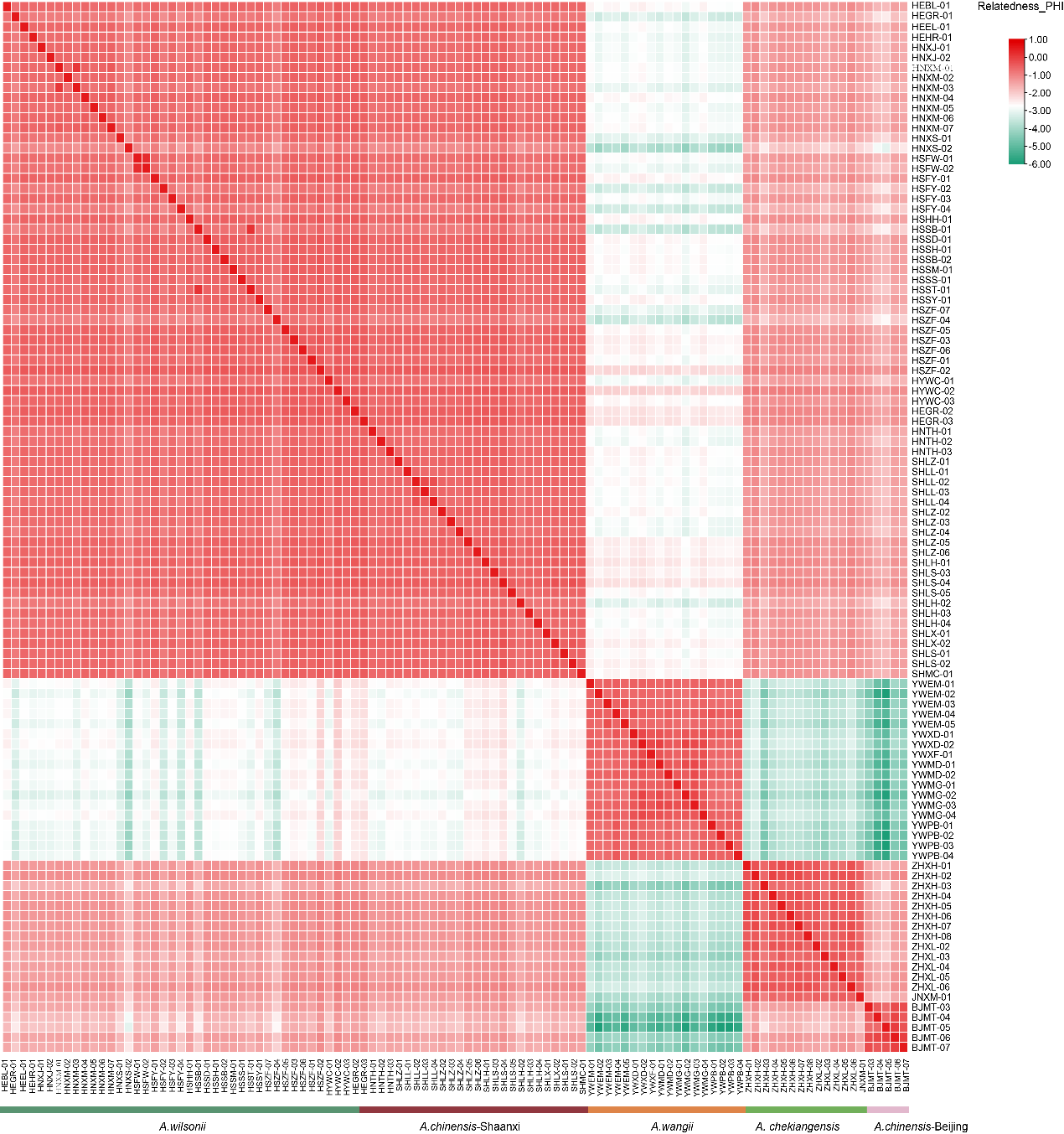


**Supplementary Figure 18.** Heatmap of individuals relatedness.

**Supplementary Table 1.** List of re-sequencing buckeye accessions

| **Sample ID** | **Species** | **Location** | **Longitude** | **Latitude** | **Altitude/m** | **Clean Reads (Gb)** | **Depth (fold/0.5Gb)** | **Mapping rate (%)** |
| --- | --- | --- | --- | --- | --- | --- | --- | --- |
| HSZF-01 | *A. wilsonii* | Hubei province, China | 109.72 | 31.89 | 1122.74 | 22405380 | 5.2 | 96.46% |
| HSZF-02 | *A. wilsonii* | Hubei province, China | 109.72 | 31.89 | 1122.74 | 27046956 | 6.162 | 94.69% |
| HSZF-03 | *A. wilsonii* | Hubei province, China | 109.69 | 31.90 | 903.88 | 26518430 | 5.855 | 91.56% |
| HSZF-04 | *A. wilsonii* | Hubei province, China | 109.66 | 31.87 | 1093.72 | 21491090 | 4.754 | 92.00% |
| HSZF-05 | *A. wilsonii* | Hubei province, China | 109.66 | 31.87 | 1093.72 | 22753440 | 5.178 | 94.54% |
| HSZF-06 | *A. wilsonii* | Hubei province, China | 109.67 | 31.86 | 917.61 | 24487270 | 5.394 | 91.54% |
| HSZF-07 | *A. wilsonii* | Hubei province, China | 109.64 | 31.78 | 1008.60 | 22778770 | 3.962 | 74.62% |
| HSFY-01 | *A. wilsonii* | Hubei province, China | 110.72 | 31.90 | 790.12 | 22445300 | 5.253 | 97.17% |
| HSFY-02 | *A. wilsonii* | Hubei province, China | 110.55 | 31.88 | 998.92 | 22012974 | 5.166 | 97.52% |
| HSFY-03 | *A. wilsonii* | Hubei province, China | 110.54 | 31.87 | 1079.44 | 21922776 | 4.484 | 85.03% |
| HSFY-04 | *A. wilsonii* | Hubei province, China | 110.64 | 31.86 | 1209.42 | 20772702 | 4.937 | 98.78% |
| HSFW-01 | *A. wilsonii* | Hubei province, China | 110.91 | 31.97 | 467.61 | 27407010 | 6.129 | 92.86% |
| HSFW-02 | *A. wilsonii* | Hubei province, China | 110.91 | 31.97 | 477.26 | 22689440 | 5.119 | 93.71% |
| HSSB-01 | *A. wilsonii* | Hubei province, China | 110.60 | 31.75 | 1073.48 | 24539770 | 5.724 | 96.27% |
| HSSB-02 | *A. wilsonii* | Hubei province, China | 110.55 | 31.78 | 1267.60 | 23609454 | 5.047 | 88.77% |
| HSST-01 | *A. wilsonii* | Hubei province, China | 110.63 | 31.76 | 959.56 | 22087832 | 5.245 | 97.99% |
| HSSM-01 | *A. wilsonii* | Hubei province, China | 110.61 | 31.81 | 1140.79 | 25745748 | 5.282 | 85.40% |
| HSSY-01 | *A. wilsonii* | Hubei province, China | 110.65 | 31.75 | 932.05 | 23407958 | 4.808 | 85.26% |
| HSSD-01 | *A. wilsonii* | Hubei province, China | 110.50 | 31.80 | 1628.76 | 21463056 | 5.015 | 97.09% |
| HSSH-01 | *A. wilsonii* | Hubei province, China | 110.66 | 31.69 | 1063.24 | 23988714 | 5.277 | 91.53% |
| HSSS-01 | *A. wilsonii* | Hubei province, China | 110.60 | 31.66 | 899.82 | 21570538 | 4.666 | 89.84% |
| HSHH-01 | *A. wilsonii* | Hubei province, China | 110.42 | 31.67 | 1615.26 | 23823898 | 5.304 | 92.60% |
| HEBY-01 | *A. wilsonii* | Hubei province, China | 110.35 | 31.28 | 914.58 | 21437356 | 5 | 96.83% |
| HEEL-01 | *A. wilsonii* | Hubei province, China | 109.54 | 30.25 | 896.12 | 22816910 | 4.006 | 73.07% |
| HEHR-01 | *A. wilsonii* | Hubei province, China | 110.13 | 29.92 | 1089.95 | 20213244 | 4.258 | 87.67% |
| HYWC-01 | *A. wilsonii* | Hubei province, China | 110.92 | 30.17 | 862.25 | 21512066 | 4.701 | 90.90% |
| HYWC-02 | *A. wilsonii* | Hubei province, China | 110.91 | 30.18 | 990.04 | 30523272 | 6.75 | 92.38% |
| HYWC-03 | *A. wilsonii* | Hubei province, China | 110.91 | 30.15 | 815.38 | 27395044 | 5.219 | 79.20% |
| HEGR-01 | *A. wilsonii* | Hubei province, China | 114.66 | 30.53 | 8.36 | 21047818 | 4.919 | 97.27% |
| HEGR-02 | *A. wilsonii* | Hubei province, China | 114.66 | 30.53 | 12.90 | 26191692 | 6.235 | 98.75% |
| HEGR-03 | *A. chinensis-Shaanxi* | Hubei province, China | 114.66 | 30.53 | 12.10 | 25409520 | 6.063 | 99.02% |
| HNTH-01 | *A. chinensis- Shaanxi* | Henan province, China | 113.29 | 32.41 | 220.00 | 22716734 | 5.377 | 98.81% |
| HNTH-02 | *A. chinensis-* *Shaanxi* | Henan province, China | 113.33 | 32.42 | 230.00 | 23010098 | 5.297 | 96.91% |
| HNTH-03 | *A. chinensis- Shaanxi* | Henan province, China | 113.30 | 32.41 | 207.14 | 23724996 | 5.586 | 98.48% |
| HNXS-01 | *A. wilsonii* | Henan province, China | 111.46 | 33.47 | 340.41 | 25596992 | 5.908 | 97.68% |
| HNXS-02 | *A. wilsonii* | Henan province, China | 111.46 | 33.47 | 338.99 | 23518922 | 5.576 | 98.85% |
| HNXJ-01 | *A. wilsonii* | Henan province, China | 111.51 | 33.52 | 527.84 | 20546882 | 4.77 | 96.89% |
| HNXJ-02 | *A. wilsonii* | Henan province, China | 111.50 | 33.52 | 466.54 | 22019038 | 5.179 | 98.10% |
| HNXM-01 | *A. wilsonii* | Henan province, China | 111.47 | 33.66 | 750.05 | 24742300 | 5.059 | 85.33% |
| HNXM-02 | *A. wilsonii* | Henan province, China | 111.46 | 33.66 | 749.26 | 26418242 | 6.256 | 98.83% |
| HNXM-03 | *A. wilsonii* | Henan province, China | 111.46 | 33.66 | 748.47 | 27513756 | 6.532 | 98.97% |
| HNXM-04 | *A. wilsonii* | Henan province, China | 111.46 | 33.66 | 747.69 | 27070054 | 6.368 | 98.59% |
| HNXM-05 | *A. wilsonii* | Henan province, China | 111.46 | 33.65 | 748.69 | 28517332 | 6.605 | 97.64% |
| HNXM-06 | *A. wilsonii* | Henan province, China | 111.46 | 33.64 | 730.26 | 22126744 | 5.269 | 99.15% |
| HNXM-07 | *A. wilsonii* | Henan province, China | 111.43 | 33.59 | 493.44 | 21731872 | 5.175 | 99.12% |
| SHLZ-01 | *A. chinensis-Shaanxi* | Shaanxi province, China | 106.97 | 33.56 | 774.06 | 22734526 | 5.266 | 96.60% |
| SHLZ-02 | *A. chinensis- Shaanxi* | Shaanxi province, China | 106.95 | 33.58 | 837.30 | 22755040 | 5.312 | 97.77% |
| SHLZ-03 | *A. chinensis-* *Shaanxi* | Shaanxi province, China | 106.93 | 33.61 | 930.48 | 20600000 | 4.657 | 94.87% |
| SHLZ-04 | *A. chinensis-Shaanxi* | Shaanxi province, China | 106.92 | 33.61 | 987.06 | 24221606 | 5.688 | 98.33% |
| SHLZ-05 | *A. chinensis- Shaanxi* | Shaanxi province, China | 106.92 | 33.62 | 956.92 | 24797480 | 5.793 | 97.38% |
| SHLZ-06 | *A. chinensis-* *Shaanxi* | Shaanxi province, China | 106.90 | 33.64 | 997.96 | 24930052 | 5.75 | 96.48% |
| SHLL-01 | *A. chinensis-Shaanxi* | Shaanxi province, China | 106.84 | 33.68 | 1236.39 | 22546776 | 5.3 | 98.48% |
| SHLL-02 | *A. chinensis- Shaanxi* | Shaanxi province, China | 106.84 | 33.68 | 1233.60 | 21323340 | 5.012 | 97.76% |
| SHLL-03 | *A. chinensis- Shaanxi* | Shaanxi province, China | 106.84 | 33.68 | 1235.60 | 20893734 | 4.77 | 94.96% |
| SHLL-04 | *A. chinensis-Shaanxi* | Shaanxi province, China | 106.83 | 33.68 | 1240.41 | 24041460 | 5.517 | 95.68% |
| SHMC-01 | *A. chinensis-Shaanxi* | Shaanxi province, China | 106.46 | 33.19 | 585.87 | 23120608 | 5.133 | 92.73% |
| SHLH-01 | *A. chinensis-Shaanxi* | Shaanxi province, China | 106.46 | 33.22 | 610.18 | 23985268 | 5.656 | 98.08% |
| SHLH-02 | *A. chinensis- Shaanxi* | Shaanxi province, China | 106.43 | 33.23 | 606.53 | 20400066 | 4.676 | 96.09% |
| SHLH-03 | *A. chinensis-* *Shaanxi* | Shaanxi province, China | 106.43 | 33.25 | 605.23 | 21671896 | 5.077 | 98.17% |
| SHLH-04 | *A. chinensis-Shaanxi* | Shaanxi province, China | 106.41 | 33.28 | 636.64 | 21132068 | 4.978 | 98.21% |
| SHLX-01 | *A. chinensis- Shaanxi* | Shaanxi province, China | 106.15 | 33.33 | 709.73 | 22287398 | 5.265 | 98.84% |
| SHLX-02 | *A. chinensis-* *Shaanxi* | Shaanxi province, China | 106.15 | 33.33 | 616.58 | 23792460 | 5.63 | 98.89% |
| SHLS-01 | *A. chinensis-Shaanxi* | Shaanxi province, China | 106.04 | 33.20 | 552.87 | 19634648 | 4.653 | 98.39% |
| SHLS-02 | *A. chinensis- Shaanxi* | Shaanxi province, China | 106.03 | 33.20 | 583.72 | 25075642 | 5.907 | 98.00% |
| SHLS-03 | *A. chinensis-* *Shaanxi* | Shaanxi province, China | 106.02 | 33.20 | 627.56 | 24270058 | 5.717 | 98.66% |
| SHLS-04 | *A. chinensis-Shaanxi* | Shaanxi province, China | 106.00 | 33.20 | 644.87 | 26052462 | 5.772 | 92.31% |
| SHLS-05 | *A. chinensis- Shaanxi* | Shaanxi province, China | 105.98 | 33.21 | 619.26 | 24315916 | 5.71 | 98.33% |
| BJMT-01 | *A. chinensis-Beijing* | Beijing, China | 116.02 | 39.90 | 327.03 | 21135994 | 5.03 | 99.10% |
| BJMT-02 | *A. chinensis-Beijing* | Beijing, China | 116.03 | 39.90 | 337.53 | 22046378 | 5.219 | 98.88% |
| BJMT-03 | *A. chinensis-Beijing* | Beijing, China | 116.03 | 39.90 | 336.95 | 24443324 | 5.556 | 97.75% |
| BJMT-04 | *A. chinensis-Beijing* | Beijing, China | 116.03 | 39.90 | 341.34 | 24068006 | 5.712 | 99.01% |
| BJMT-05 | *A. chinensis-Beijing* | Beijing, China | 116.03 | 39.90 | 334.14 | 25342510 | 5.976 | 98.54% |
| JNXM-01 | *A. chekiangensis* | Jiangsu, China | 118.83 | 32.06 | 48.49 | 20108100 | 4.764 | 98.85% |
| ZHXL-02 | *A. chekiangensis* | Zhejiang, China | 120.10 | 30.24 | 52.00 | 20119134 | 4.636 | 96.40% |
| ZHXL-03 | *A. chekiangensis* | Zhejiang, China | 120.10 | 30.24 | 46.32 | 22513318 | 5.336 | 98.61% |
| ZHXL-04 | *A. chekiangensis* | Zhejiang, China | 120.10 | 30.24 | 54.97 | 24362216 | 5.744 | 98.08% |
| ZHXL-05 | *A. chekiangensis* | Zhejiang, China | 120.10 | 30.24 | 53.77 | 21900240 | 5.154 | 97.97% |
| ZHXL-06 | *A. chekiangensis* | Zhejiang, China | 120.10 | 30.24 | 54.15 | 19699544 | 4.626 | 97.78% |
| ZHXH-01 | *A. chekiangensis* | Zhejiang, China | 120.13 | 30.21 | 62.57 | 19677592 | 4.68 | 98.71% |
| ZHXH-02 | *A. chekiangensis* | Zhejiang, China | 120.13 | 30.21 | 62.14 | 24306236 | 5.758 | 98.79% |
| ZHXH-03 | *A. chekiangensis* | Zhejiang, China | 120.13 | 30.21 | 59.21 | 23670118 | 5.588 | 98.65% |
| ZHXH-04 | *A. chekiangensis* | Zhejiang, China | 120.13 | 30.21 | 66.07 | 22840384 | 5.316 | 97.30% |
| ZHXH-05 | *A. chekiangensis* | Zhejiang, China | 120.13 | 30.21 | 62.34 | 22763414 | 5.337 | 97.91% |
| ZHXH-06 | *A. chekiangensis* | Zhejiang, China | 120.13 | 30.21 | 61.61 | 24841874 | 5.639 | 94.60% |
| ZHXH-07 | *A. chekiangensis* | Zhejiang, China | 120.13 | 30.21 | 69.16 | 23175332 | 5.423 | 97.39% |
| ZHXH-08 | *A. chekiangensis* | Zhejiang, China | 120.13 | 30.22 | 26.21 | 25829914 | 6.012 | 97.36% |
| YWEM-01 | *A. wangii* | Yunnan, China | 103.97 | 23.31 | 1949.27 | 22816686 | 5.091 | 93.76% |
| YWEM-02 | *A. wangii* | Yunnan, China | 103.97 | 23.31 | 1850.27 | 22913698 | 3.971 | 73.21% |
| YWEM-03 | *A. wangii* | Yunnan, China | 103.97 | 23.30 | 1881.22 | 19771404 | 4.371 | 92.84% |
| YWEM-04 | *A. wangii* | Yunnan, China | 103.98 | 23.31 | 1822.29 | 26150220 | 5.307 | 85.36% |
| YWEM-05 | *A. wangii* | Yunnan, China | 103.98 | 23.31 | 1853.27 | 27036526 | 5.061 | 78.90% |
| YWXD-01 | *A. wangii* | Yunnan, China | 104.85 | 23.37 | 1311.00 | 23376248 | 5.324 | 95.98% |
| YWXD-02 | *A. wangii* | Yunnan, China | 104.85 | 23.37 | 1313.00 | 22889494 | 5.12 | 94.03% |
| YWXF-01 | *A. wangii* | Yunnan, China | 104.79 | 23.38 | 1536.00 | 24575992 | 5.594 | 95.98% |
| YWMD-01 | *A. wangii* | Yunnan, China | 104.26 | 23.05 | 1468.00 | 24888454 | 5.211 | 87.91% |
| YWMD-01 | *A. wangii* | Yunnan, China | 104.26 | 23.05 | 1468.00 | 24582884 | 5.255 | 89.91% |
| YWMG-01 | *A. wangii* | Yunnan, China | 104.00 | 22.86 | 1776.00 | 20671002 | 4.471 | 91.25% |
| YWMG-02 | *A. wangii* | Yunnan, China | 104.00 | 22.86 | 1776.00 | 25761790 | 5.485 | 89.59% |
| YWMG-03 | *A. wangii* | Yunnan, China | 104.00 | 22.86 | 1817.00 | 24161860 | 5.287 | 92.12% |
| YWMG-04 | *A. wangii* | Yunnan, China | 103.99 | 22.86 | 1722.00 | 26059542 | 5.687 | 91.87% |
| YWPB-01 | *A. wangii* | Yunnan, China | 103.80 | 22.97 | 1706.00 | 22130384 | 4.494 | 85.63% |
| YWPB-02 | *A. wangii* | Yunnan, China | 103.80 | 22.97 | 1718.00 | 21581556 | 3.975 | 77.63% |
| YWPB-03 | *A. wangii* | Yunnan, China | 103.80 | 22.97 | 1705.00 | 20677038 | 4.523 | 92.35% |
| YWPB-04 | *A. wangii* | Yunnan, China | 103.80 | 22.97 | 1710.00 | 25678876 | 4.595 | 75.52% |

**Supplementary Table 2.**K-mer analysis

| **Sample** | **K-mer** | **K-mer**  **Number** | **K-mer Depth** | **Genome Size（M）** | **Data Size（G）** | **X** | **Heterozygous Ratio (%)** | **Repeat (%)** |
| --- | --- | --- | --- | --- | --- | --- | --- | --- |
| *A. wilsonii* | 19 | 24,927,287,462 | 44.93 | 552.87 | 38.59 | 69.8 | 1.22 | 53.91 |

**Supplementary** **Table 3.** The statistics of sequencing raw data from Illumina and Nanopore platforms.

| **Items** | **ONT reads** | **Illumina reads** |
| --- | --- | --- |
| Total number of reads | 3,136,320 | 425,087,626 |
| Total number of sequenced bases (bp) | 52,113,891,903 | 63,763,143,900 |
| Average reads length (bp) | 16,616.25 | 150 |
| Max reads length (bp) | 239,773 | 150 |
| N50 (bp) | 39,252 | - |

**Supplementary Table 4.** Data statistics of assembly results

| Item | *A. wilsonii* |
| --- | --- |
| Total_length (bp) | 611,221,257 |
| Total_length_without N(bp) | 611,221,250 |
| Total_number of Contig | 376 |
| GC_content (%) | 35.31 |
| Contig N50 (bp) | 3,752,046 |
| Contig N90 (bp) | 816,654 |
| Contig N50 (bp) | 28,019,856 |
| Contig N90 (bp) | 23,765,142 |

**Supplementary Table** **5.** Statistics of Hi-C data and assessment.

| **Statistics of Hi-C data** | | | |
| --- | --- | --- | --- |
| Number of clean reads | Number of bases (bp) | Clean reads rate (%) | % ≥ Q30 |
| 440,201,790 | 66,363,969,000 | 99.5 | 93.66 |
| **Statistics of mapping** | | | |
| Mapping type | | Number of reads | Ratio (%) |
| Total read pairs | | 221,213,230 | 100 |
| Mapped reads | | 536,897,669 | 92.0 |
| Unique mapped read pairs | | 75,353,070 | 34.06 |
| **Statistics of valid Hi-C data** | | | |
| Type | | Number of reads | Ratio (%) |
| Unique paired alignments | | 75,353,070 | 100 |
| Valid interaction pairs | | 29,283,408 | 38.86 |
| Dangling end pairs | | 14,154,477 | 18.78 |
| Self-cycle pairs | | 3,301,809 | 4.38 |
| Dumped pairs | | 22,493,187 | 29.85 |
| **Scaffold** | | N50 | N90 |
| Length(bp) | | 28,019,856 | 23,765,142 |

**Supplementary Table 6.** The length of chromosome by Hi-C assembly.

| **ID** | **Length(bp)** | **Contig_Num** |
| --- | --- | --- |
| Chr01 | 28,019,856 | 33 |
| Chr02 | 35,040,876 | 17 |
| Chr03 | 30,592,251 | 4 |
| Chr04 | 27,810,625 | 13 |
| Chr05 | 26,361,240 | 15 |
| Chr06 | 25,453,655 | 18 |
| Chr07 | 25,780,122 | 16 |
| Chr08 | 25,529,906 | 16 |
| Chr09 | 24,582,930 | 18 |
| Chr10 | 23,778,762 | 14 |
| Chr11 | 23,765,142 | 23 |
| Chr12 | 34,866,348 | 28 |
| Chr13 | 25,451,329 | 12 |
| Chr14 | 23,527,542 | 20 |
| Chr15 | 37,155,894 | 22 |
| Chr16 | 34,077,853 | 19 |
| Chr17 | 32,801,097 | 23 |
| Chr18 | 32,653,897 | 17 |
| Chr19 | 31,650,177 | 11 |
| Chr20 | 30,111,747 | 22 |
| Chr0 | 32,244,108 | 15 |
| Pseudochromosomes | 579,011,249 | 361 |
| Total | 611,255,357 | 376 |

**Supplementary Table 7.** The assembly evaluation by Illumina data

| Type | Number |
| --- | --- |
| Map_rate | 98.79% |
| Average_depth | 100.98 |
| Coverage | 94.62% |

**Supplementary Table 8.** The prediction of gene structures of the *A. wilsonii* genome

| **Item** | **Number** |
| --- | --- |
| the total number of gene | 46,914.00 |
| the average of mRNA_length | 3,259.98 |
| the average cds_length of per gene | 999.35 |
| the average exon_number of per gene | 4.94 |
| the average of exon_length | 248.23 |
| the average of intron_length | 515.02 |
| the total number of exons | 231,916.00 |
| the total number of introns | 185,002.00 |
| the total intron length | 95,279,326.00 |

**Supplementary Table 9.** Quality assessment of the genome annotation of A. *wilsonii* using BUSCOs.

| **Type** | **Number** | **Percent (%)** |
| --- | --- | --- |
| Complete BUSCOs | 232 | 91.0 |
| Complete and single-copy BUSCOs | 152 | 59.6 |
| Complete and duplicated BUSCOs | 80 | 31.4 |
| Fragmented BUSCOs | 21 | 8.2 |
| Missing BUSCOs | 2 | 0.8 |
| Total BUSCO groups searched | 255 | 100.0 |

**Supplementary Table 10.** Functional annotation of predicted protein-coding genes in the *A. wilsonii*

| **Database** | **Number of genes annotated** | **Percentage (%)** |
| --- | --- | --- |
| ALL | 46914 | 100 |
| Annotation | 44210 | 94.24 |
| Uniprot | 42452 | 90.49 |
| Pfam | 35318 | 75.28 |
| GO | 34240 | 72.98 |
| KEGG | 17006 | 36.25 |
| Pathway | 10721 | 22.85 |
| Interproscan | 40732 | 86.82 |
| NR | 35821 | 76.35 |

**Supplementary Table 11.** Prediction of noncoding RNAs in the *A*. *wilsonii*

genomes.

| **Type** | | **Copy** | **Average length (bp)** | **Total length (bp)** | **% of genome** |
| --- | --- | --- | --- | --- | --- |
| ncRNA | | 4282 | 506.39 | 2168358 | 0.3547 |
| miRNA | | 302 | 121.4636 | 36682 | 0.006001 |
| tRNA | | 801 | 74.96629 | 60048 | 0.009824 |
| rRNA | | 1866 | 1030.234 | 1922416 | 0.3145 |
| snRNA | CD-box | 965 | 103.4425 | 99822 | 0.01633 |
|  | HACA-box | 84 | 124.5476 | 10462 | 0.001711 |
|  | splicing | 176 | 1476648 | 25989 | 0.004252 |

**Supplementary Table 12.** Summary statistics of the annotated transposable elements in *A. wilsonii*

| **Item** | **Subfamily** | **Number** | **Length(bp)** | **Coverage (%)** |
| --- | --- | --- | --- | --- |
| **SINE** | / | 6,599 | 1,562,249 | 0.26% |
| **LINE** | / | 27,583 | 18,267,027 | 2.99% |
| L1 |  | 19,068 | 14,648,572 | 2.40% |
| L2 |  | 2,000 | 434,725 | 0.07% |
| **LTR** | / | 194,914 | 164,983,593 | 26.99% |
| Gypsy |  | 115,999 | 98,863,117 | 16.17% |
| Copia |  | 72,886 | 61,409,065 | 10.05% |
| **DNA** | / | 120,550 | 44,007,964 | 7.20% |
| CMC-EnSpm |  | 15,623 | 5,838,605 | 0.96% |
| MuDR |  | 16,323 | 9,267,400 | 1.52% |
| PIF-Harbinger |  | 14,420 | 4,792,202 | 0.78% |
| hAT-Ac |  | 23,983 | 7,216,992 | 1.18% |
| hAT-Tip100 |  | 5,036 | 1,872,468 | 0.31% |
| **Satellite** | / | 1,070 | 134,887 | 0.02% |
| **Simple_repeat** | / | 225,486 | 13,389,403 | 2.19% |
| **Low_complexity** | / | 37,439 | 1,988,161 | 0.33% |
| Other | / | 2,120 | 818,541 | 0.13% |
| Unknown | / | 318,115 | 99,007,808 | 16.20% |
| **Total** | / | 933,876 | 332,847,051 | 54.46% |

**Supplementary Table 13.** Statistic result of clustered gene families.

| **Species** | **Genes number** | **Genes in families** | **Un clustered genes** | **Family number** | **Unique families** | **Average genes per family** | **URLs** |
| --- | --- | --- | --- | --- | --- | --- | --- |
| *Atalantia buxifolia* | 28,412 | 21,366 | 7,046 | 18,787 | 343 | 1.14 | http://citrus.hzau.edu.cn/download.php |
| *Dimocarpus longan* | 39,282 | 32,284 | 6,998 | 17,021 | 1,149 | 1.9 | http:// 10.1093/gigascience/gix023 |
| *Citrus reticulata* | 28,833 | 22,951 | 5,882 | 20,199 | 314 | 1.14 | http://citrus.hzau.edu.cn/download.php |
| *Citrus grandis* | 30,123 | 24,810 | 5,313 | 20,380 | 410 | 1.22 | http://citrus.hzau.edu.cn/download.php |
| *Citrus sinensis* | 29,406 | 24,635 | 4,771 | 20,116 | 311 | 1.22 | http://citrus.hzau.edu.cn/download.php |
| *Chrysanthemum nankingense* | 93,890 | 70,642 | 23,248 | 19,366 | 3,616 | 3.65 | https://doi.org/10.1016/j.molp.2018.10.003 |
| *Helianthus annuus* | 52,243 | 35,875 | 16,368 | 18,625 | 2,259 | 1.93 | https://www.nature.com/articles/nature22380 |
| *Arabidopsis thaliana* | 27,416 | 22,414 | 5,002 | 14,383 | 883 | 1.56 | https://www.arabidopsis.org/download_files/Genes/TAIR10_genome_release/ |
| *Vitis vinifera* | 26,346 | 18,687 | 7,659 | 14,408 | 707 | 1.3 | http://www.genoscope.cns.fr/externe/Download/Projets/Projet_ML/data/12X/ |
| *Oryza sativa* | 39,049 | 25,292 | 13,757 | 13,728 | 2,221 | 1.84 | http://rice.plantbiology.msu.edu/ |
| *Aesculus wilsonii* | 46,914 | 31,646 | 15,268 | 18,267 | 2,090 | 1.73 | The present study |

**Supplementary Table 14.** The summary table of *Fst* between different buckeye categories.

| **Population** | ***A. chinensis*-Beijing** | ***A. chekiangensis*** | ***A. wangii*** | ***A. chinensis*-Shaanxi** | ***A. wilsonii*** |
| --- | --- | --- | --- | --- | --- |
| ***A. chinensis*-Beijing** | 0 | 0.2877 | 0.5167 | 0.1465 | 0.1401 |
| ***A. chekiangensis*** | 0.2877 | 0 | 0.4902 | 0.1805 | 0.1805 |
| ***A. wangii*** | 0.5167 | 0.4902 | 0 | 0.3166 | 0.3198 |
| ***A. chinensis*-Shaanxi** | 0.1465 | 0.1805 | 0.3166 | 0 | 0.01300 |
| ***A. wilsonii*** | 0.1401 | 0.1805 | 0.3198 | 0.01300 | 0 |

**Supplementary Table 15.** The summary table of π between different buckeye categories.

| **Population** | ***A. chinensis*-Beijing** | ***A. chekiangensis*** | ***A. wangii*** | ***A. chinensis*-Shaanxi** | ***A. wilsonii*** |
| --- | --- | --- | --- | --- | --- |
| π | 0.000949831 | 0.00168544 | 0.00162189 | 0.00235386 | 0.00242091 |
